# Supplementary material for: How Does CBG Administration Affect Sphingolipid Deposition in the Liver of Insulin-Resistant Rats?
Source: Nutrients. 2023 Oct 12;15(20):4350. doi: 10.3390/nu15204350 (PMC10609522; doi:10.3390/nu15204350)

Original images for blots

The upper images represent the uncropped chemiluminescent blot image corresponding with the images placed in the manuscript.

The lower images represent the stain-free blot image of the same membrane after transfer which is used for creating multichannel image (overlaid view) to normalize data.

Loading order: control, CBG, HFHS, HFHS+CBG.

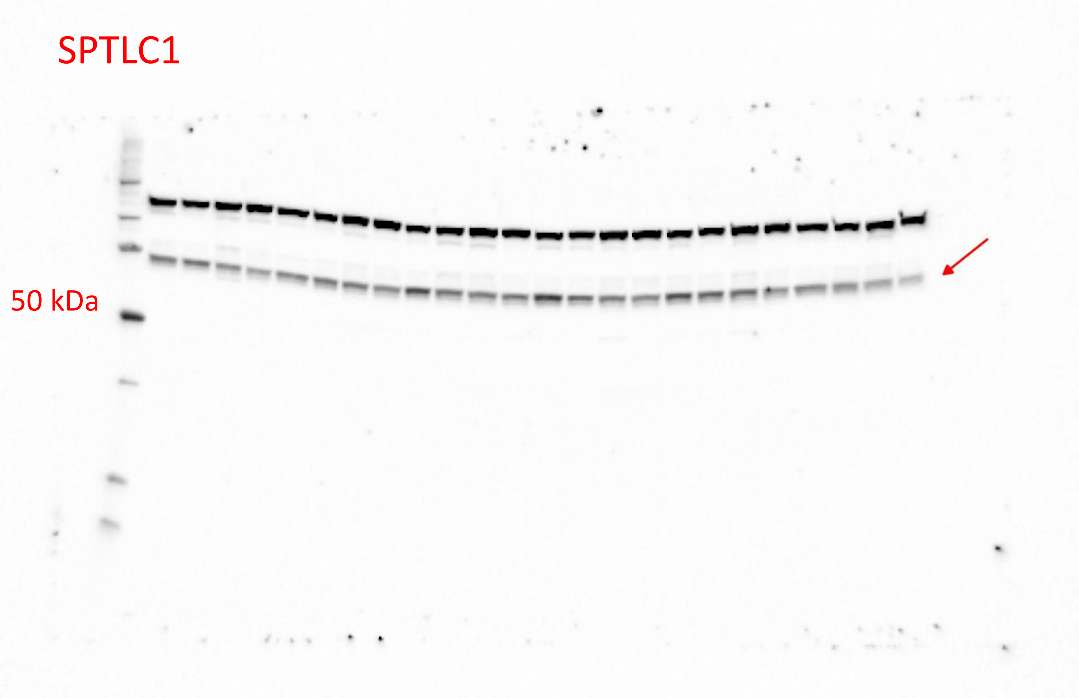
SPTLC1
50 kDa

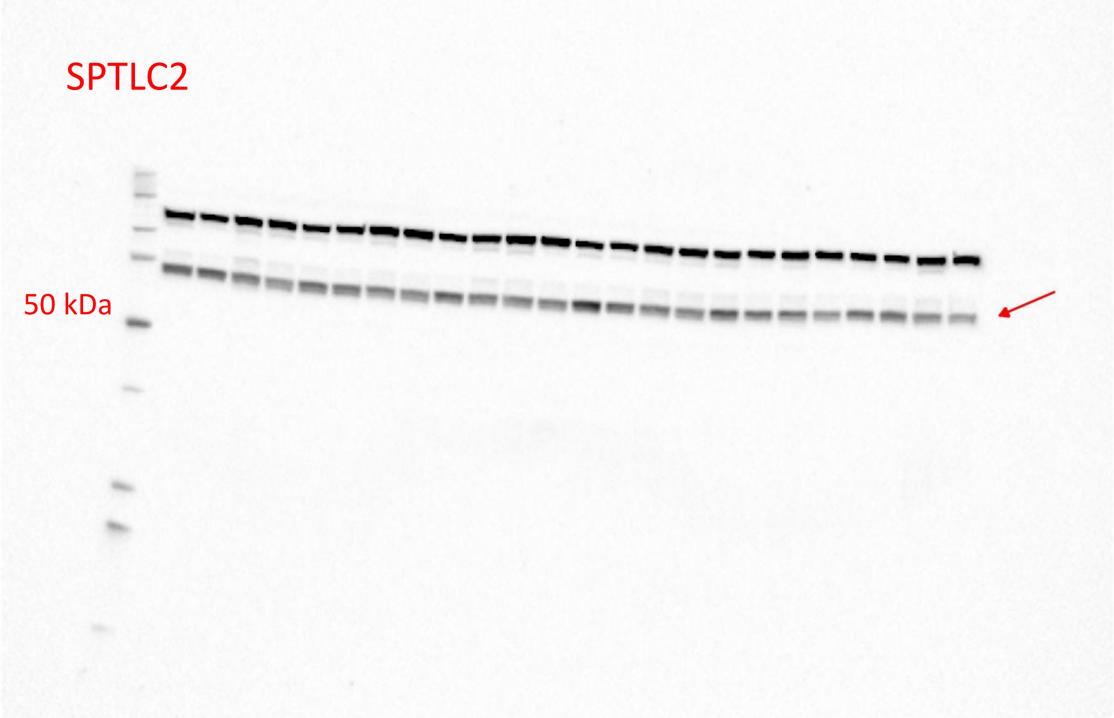
SPTLC2
50 kDa

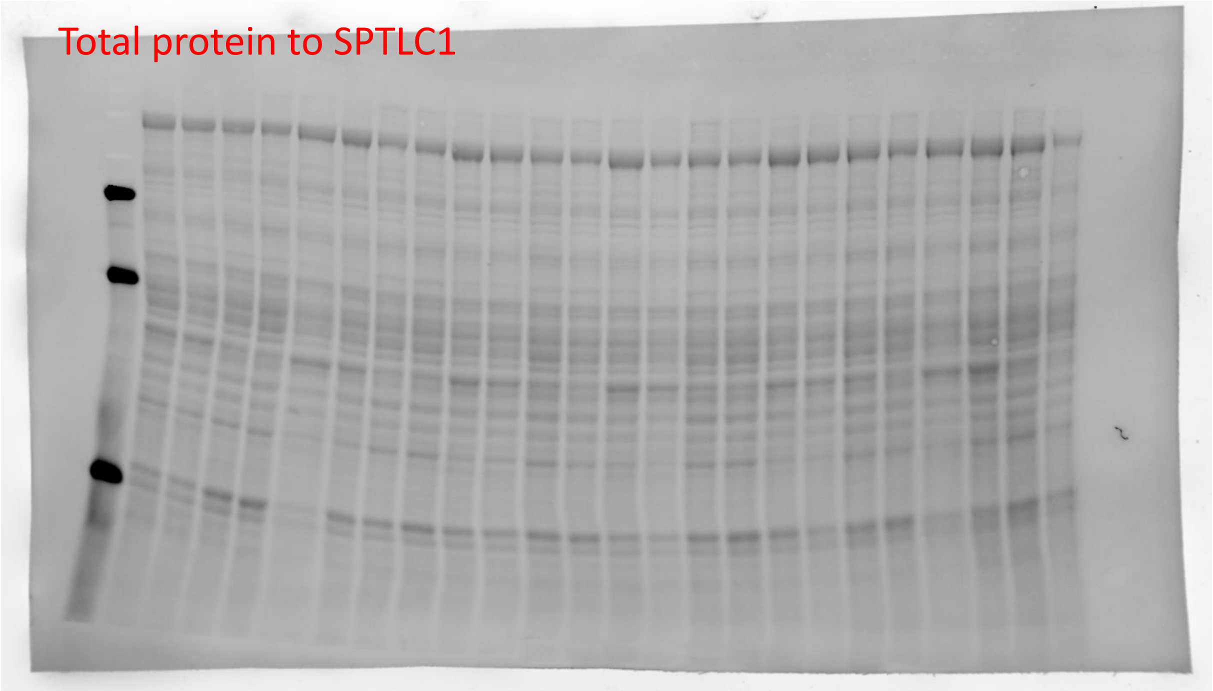
Total protein to SPTLC1

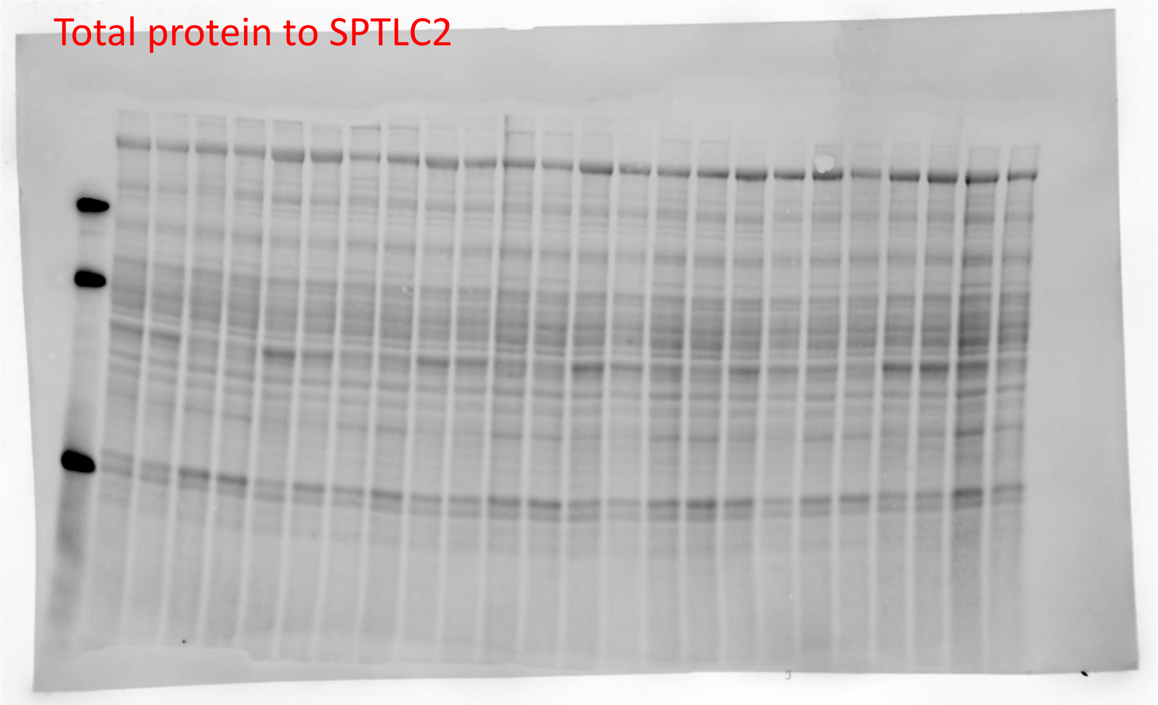
Total protein to SPTLC2

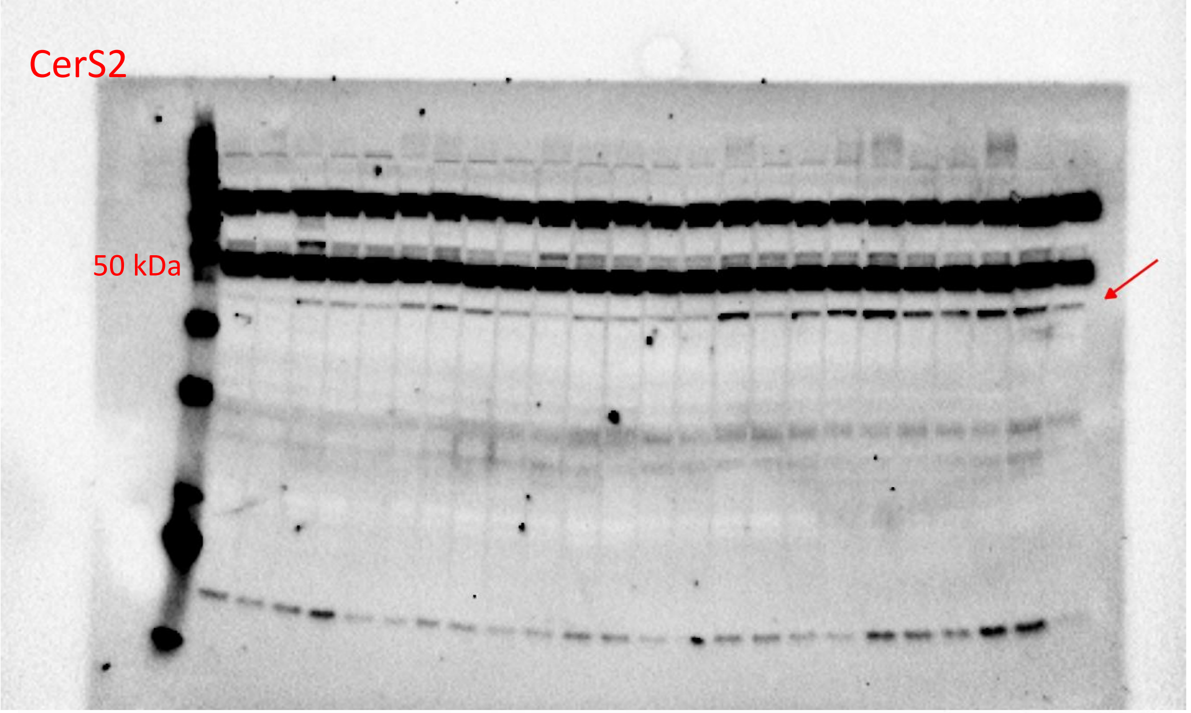
CerS2
50 kDa

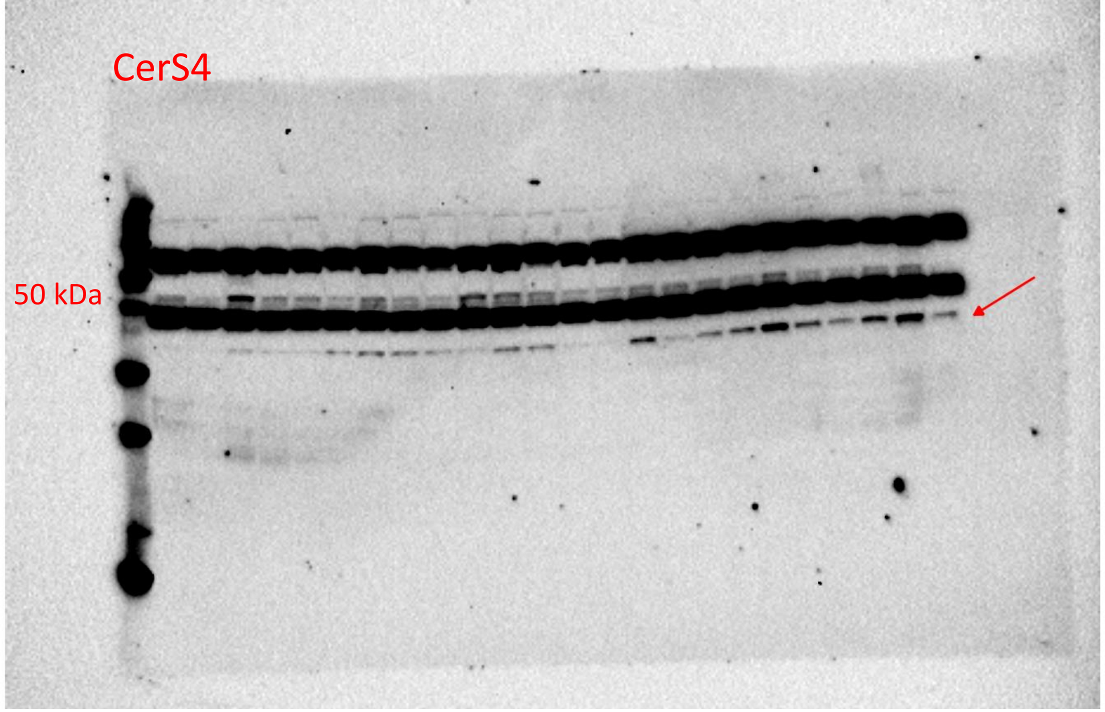
CerS4
50 kDa

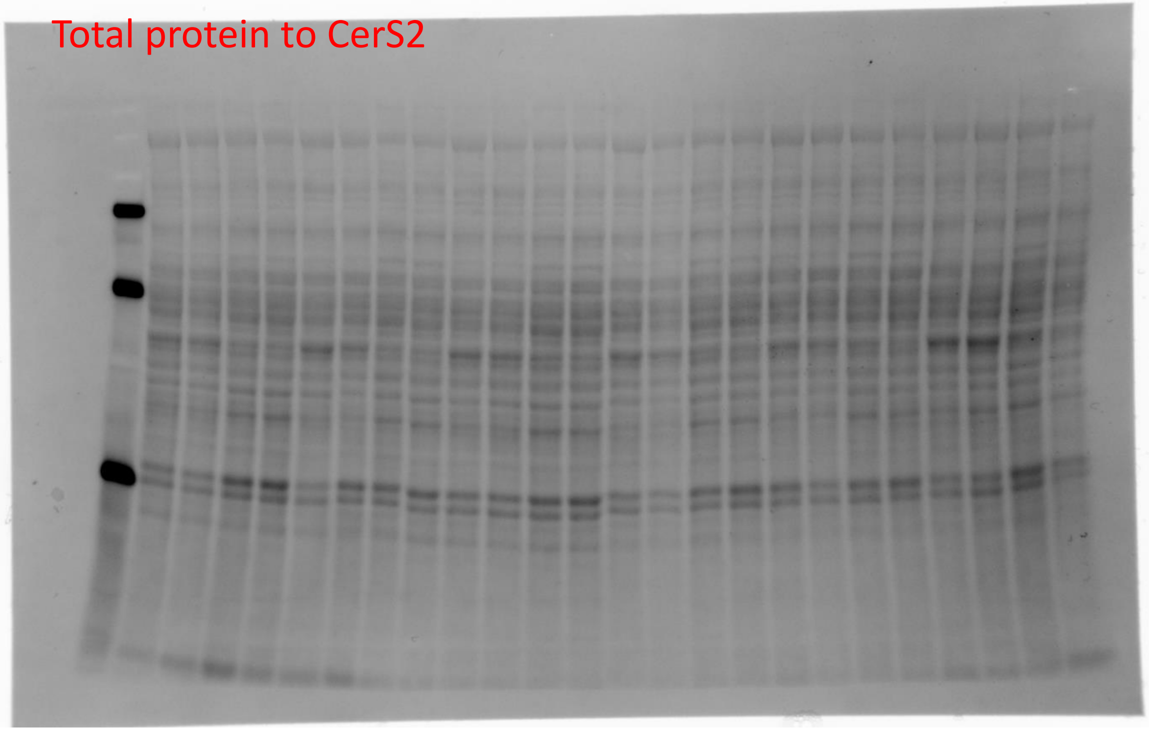
Total protein to CerS2

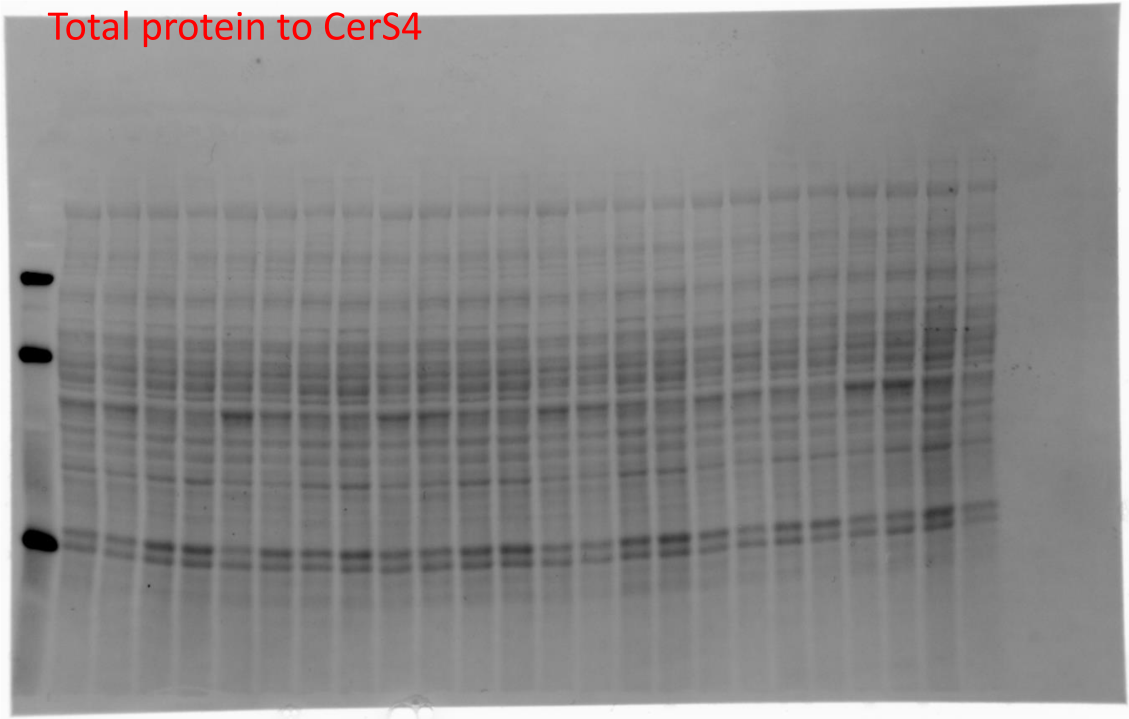
Total protein to CerS4

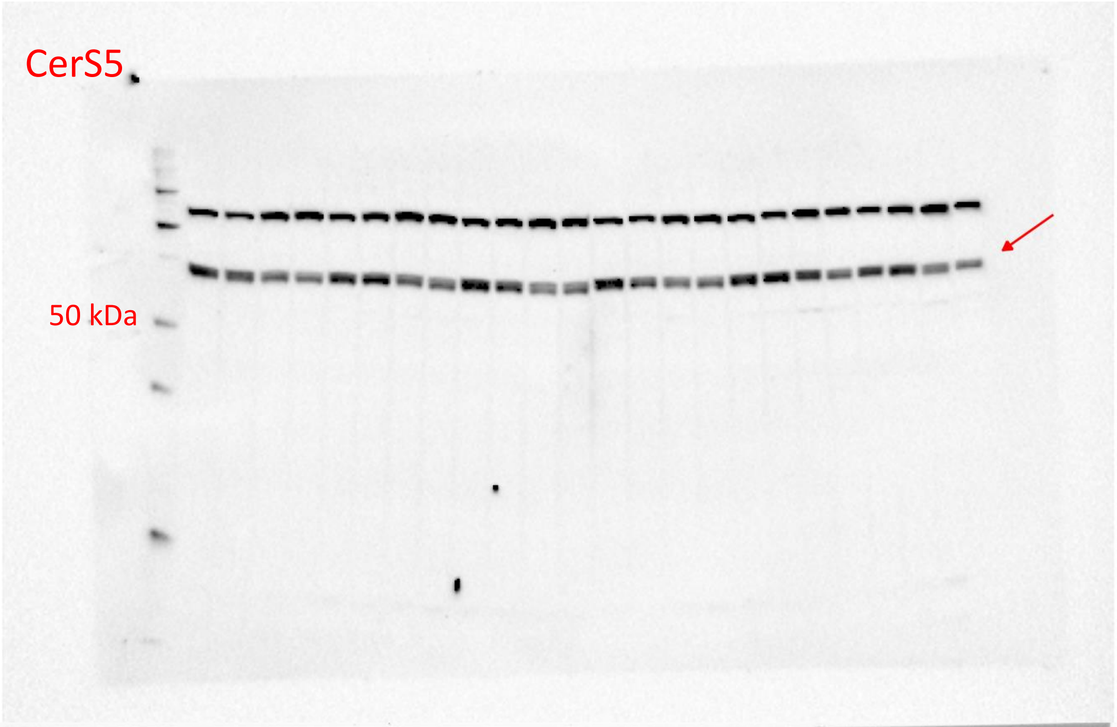
CerS5
50 kDa

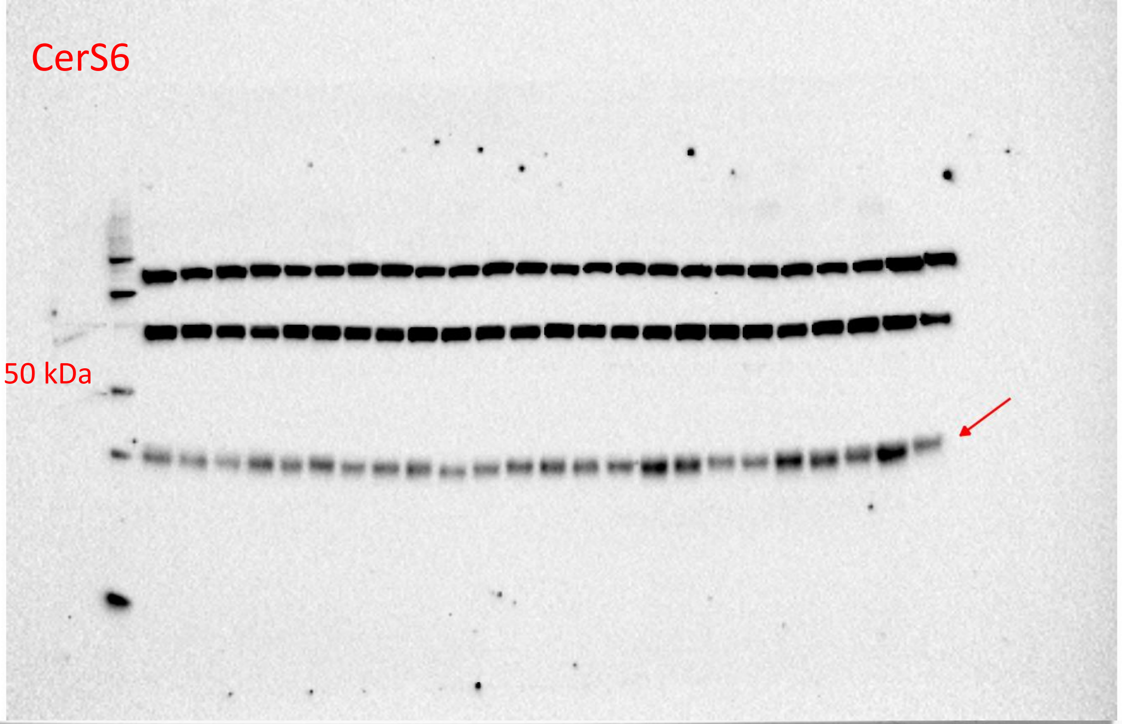
CerS6
50 kDa

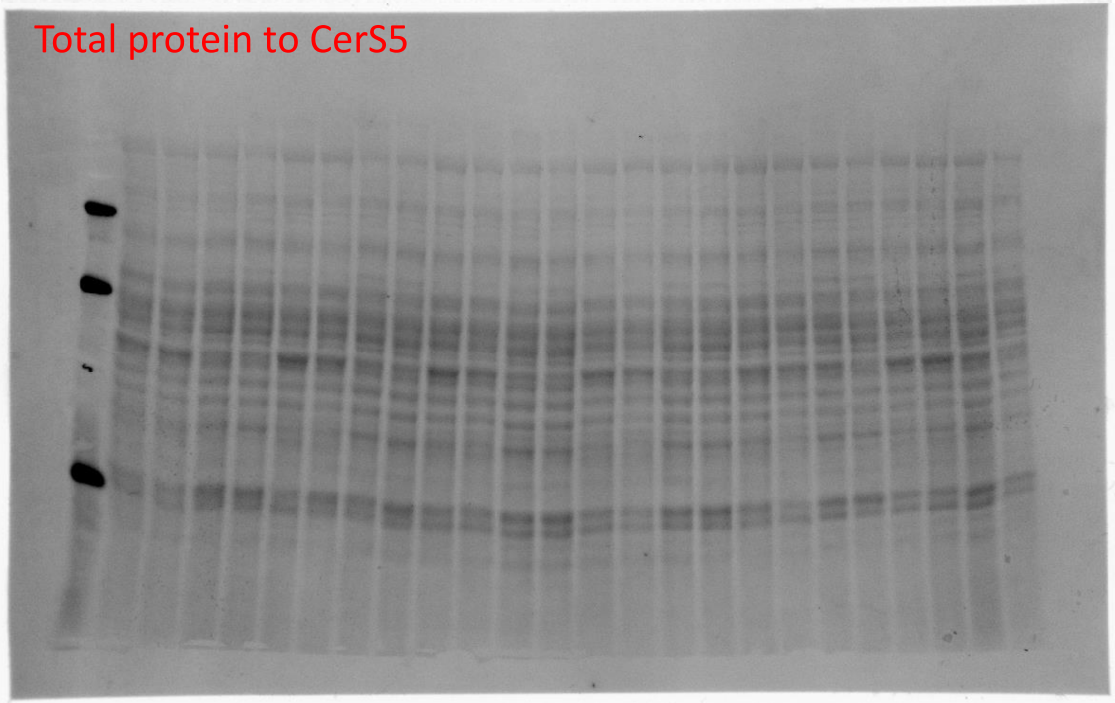
Total protein to CerS5

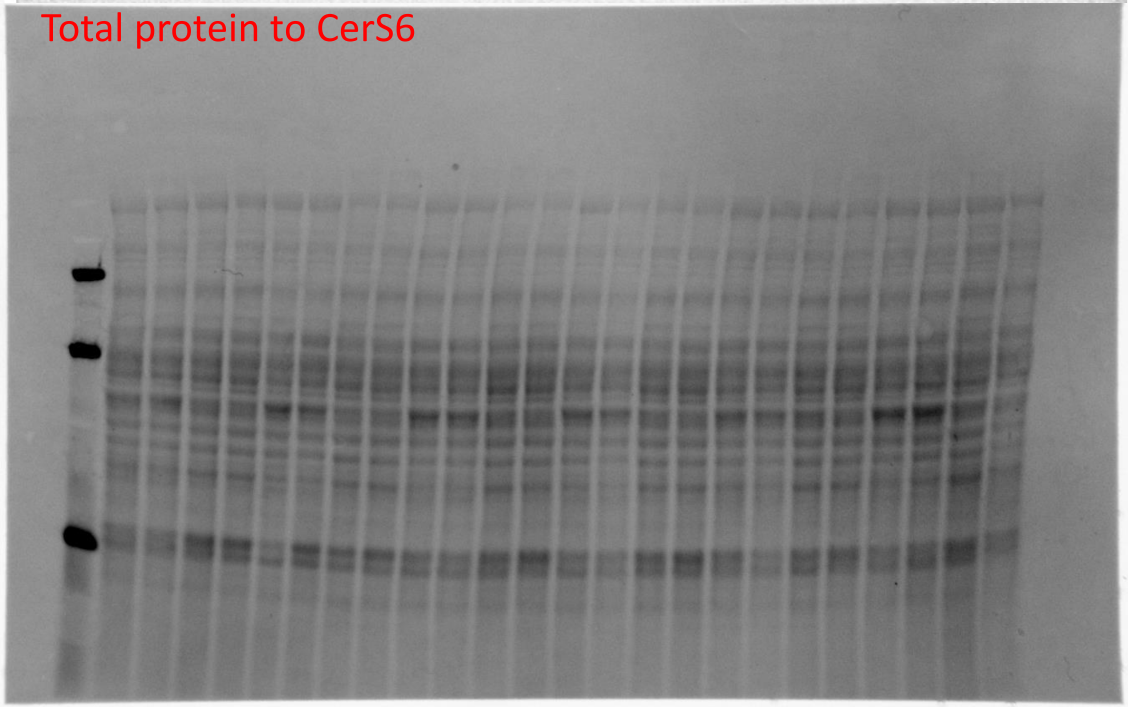
Total protein to CerS6

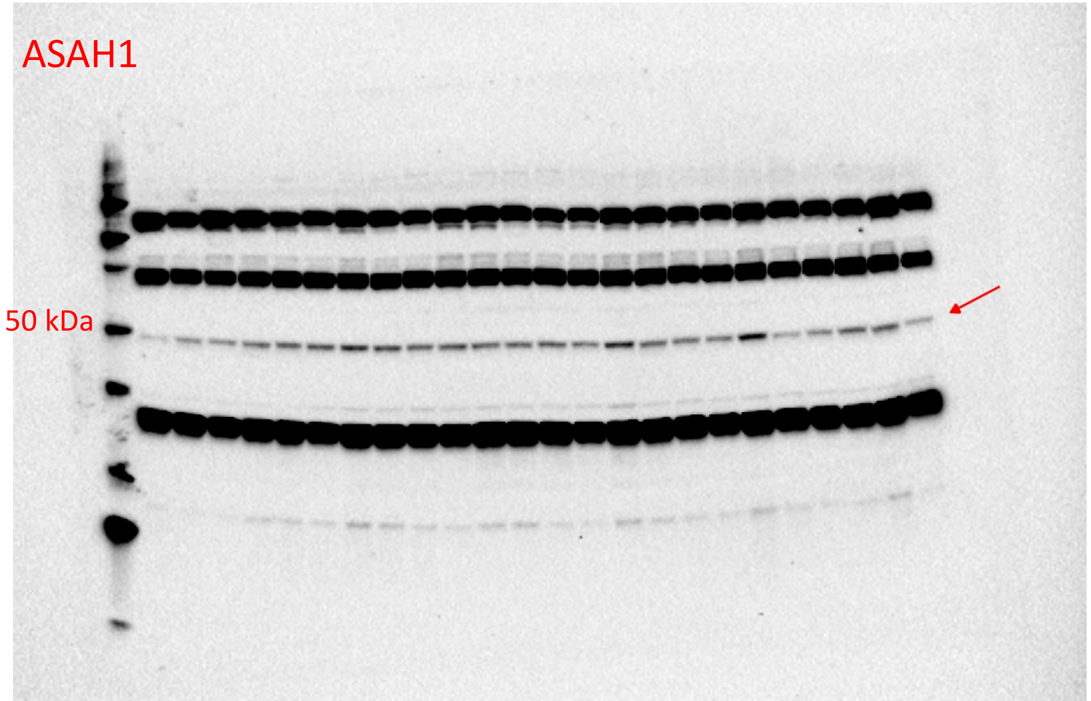
ASAH1
50 kDa

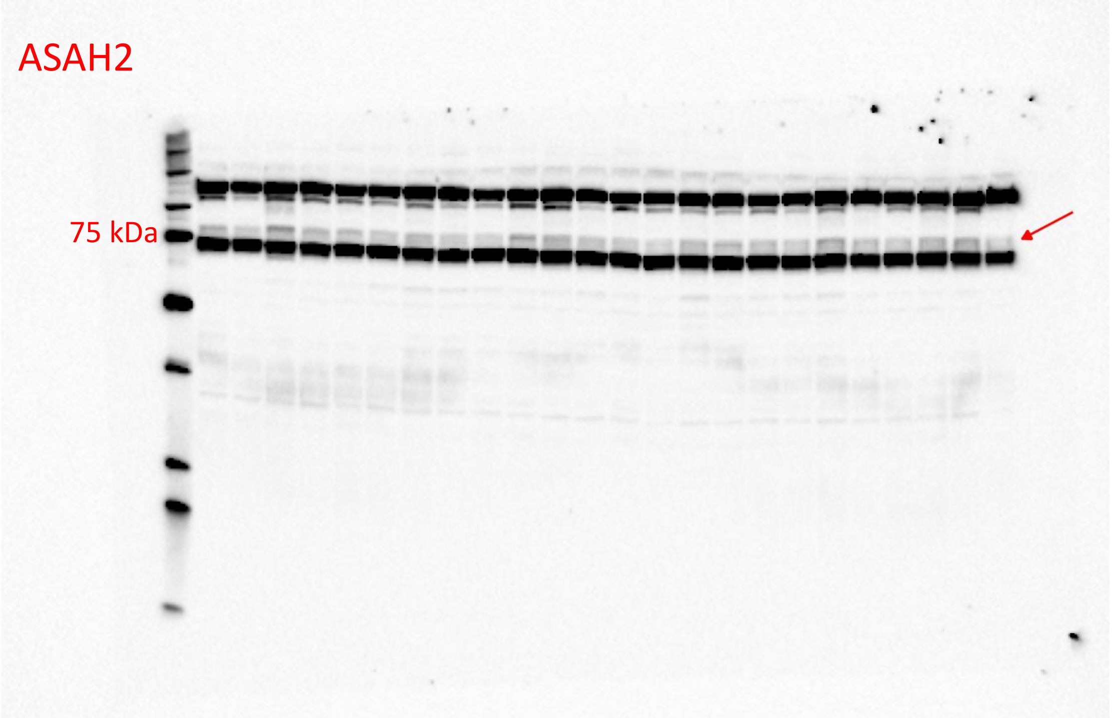
ASAH2
75 kDa

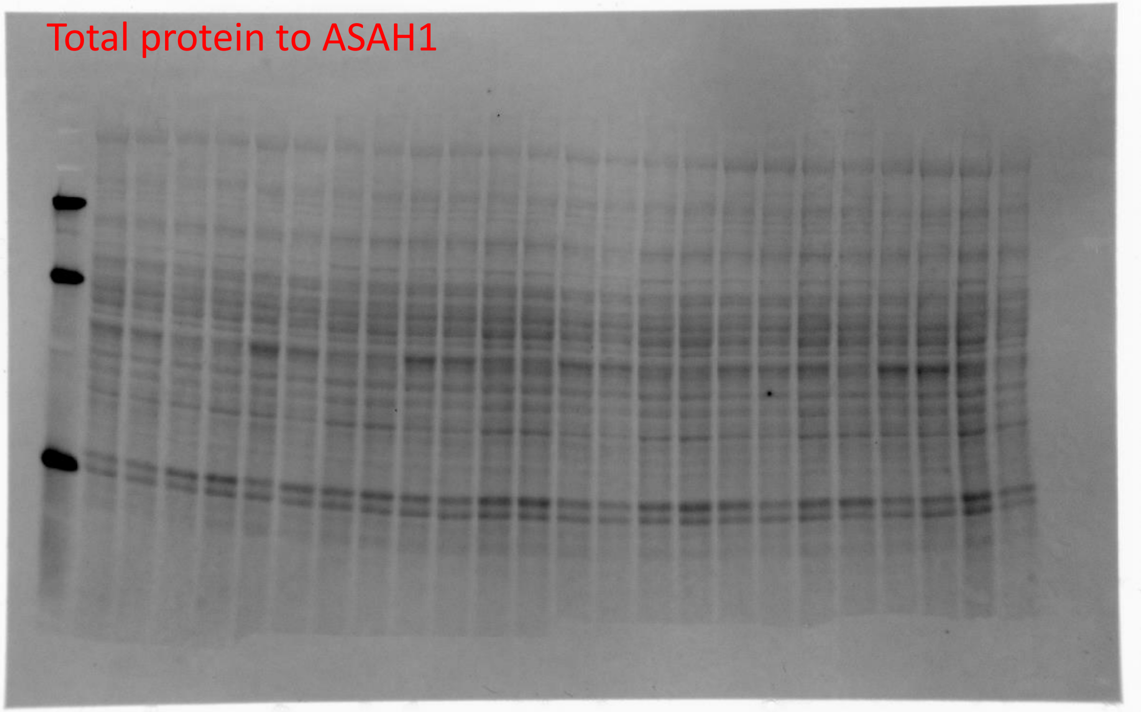
Total protein to ASAH1

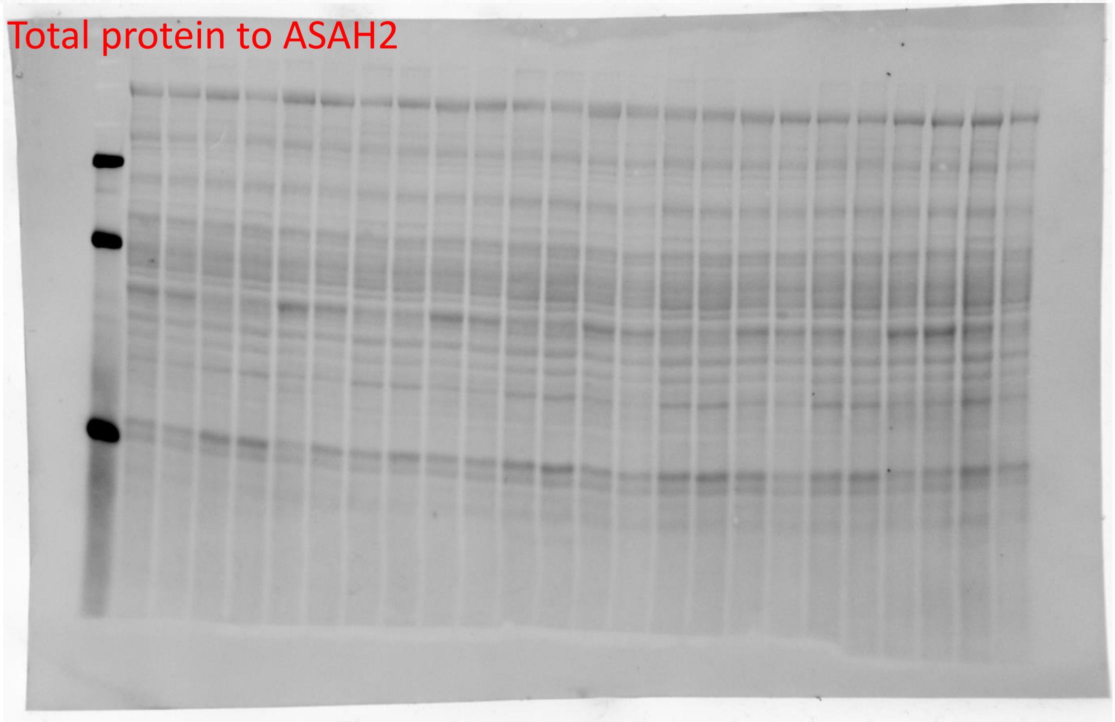
Total protein to ASAH2

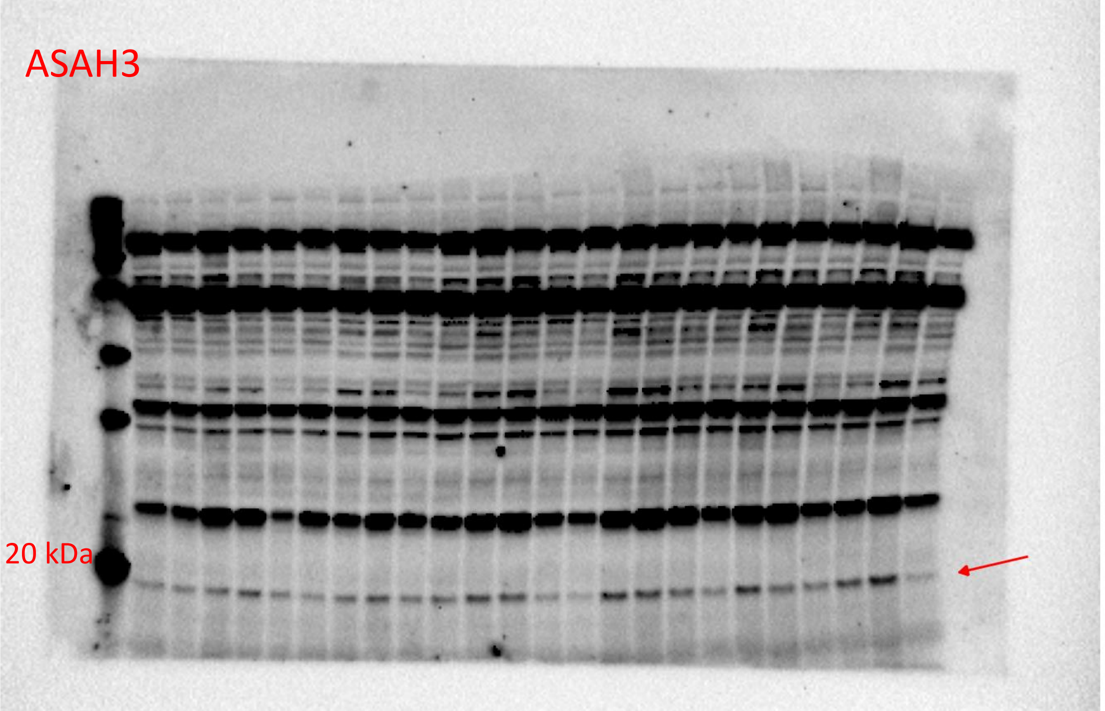
ASAH3
20 kDa

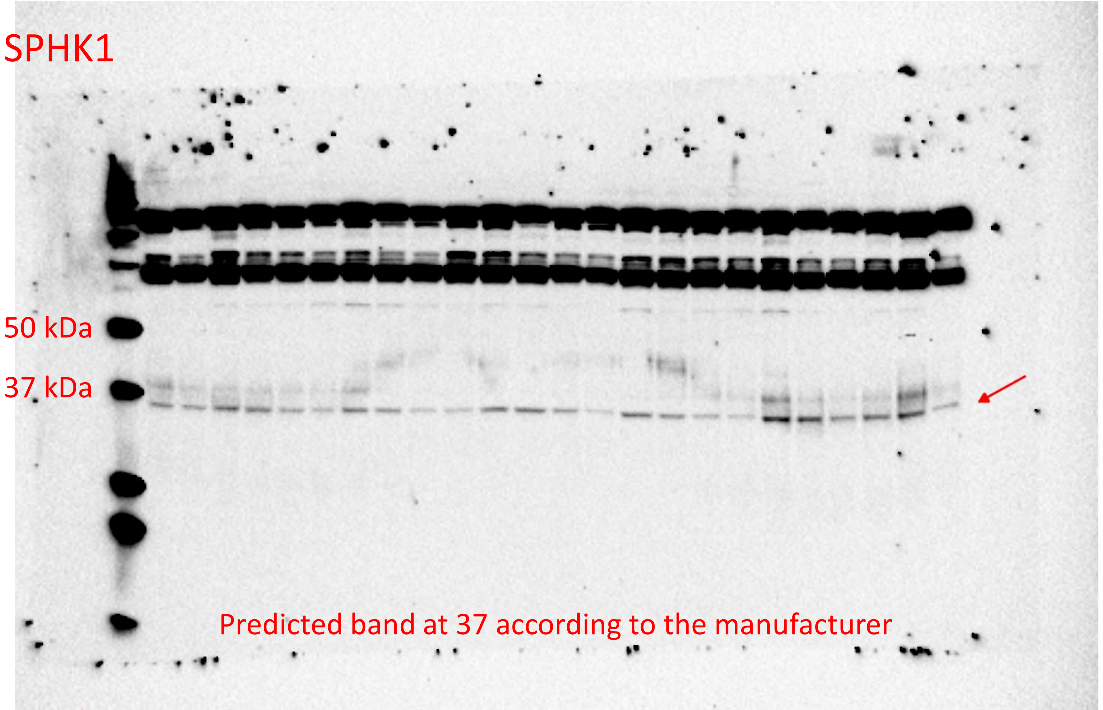
SPHK1
50 kDa
37 kDa
Predicted band at 37 according to the manufacturer

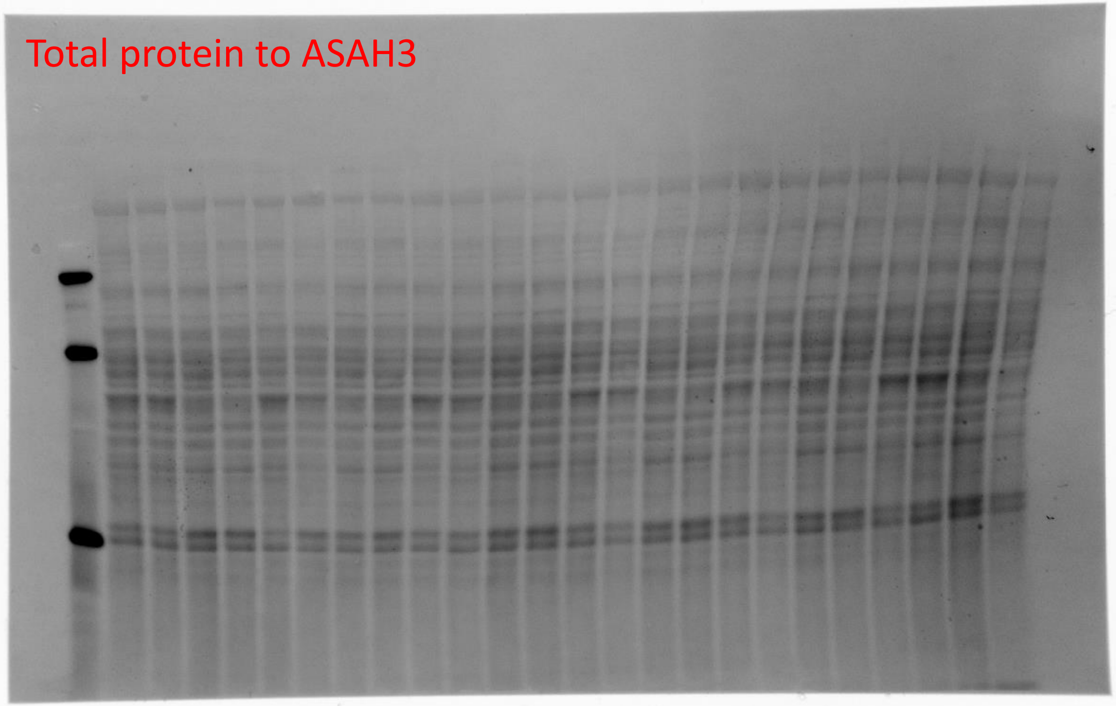
Total protein to ASAH3

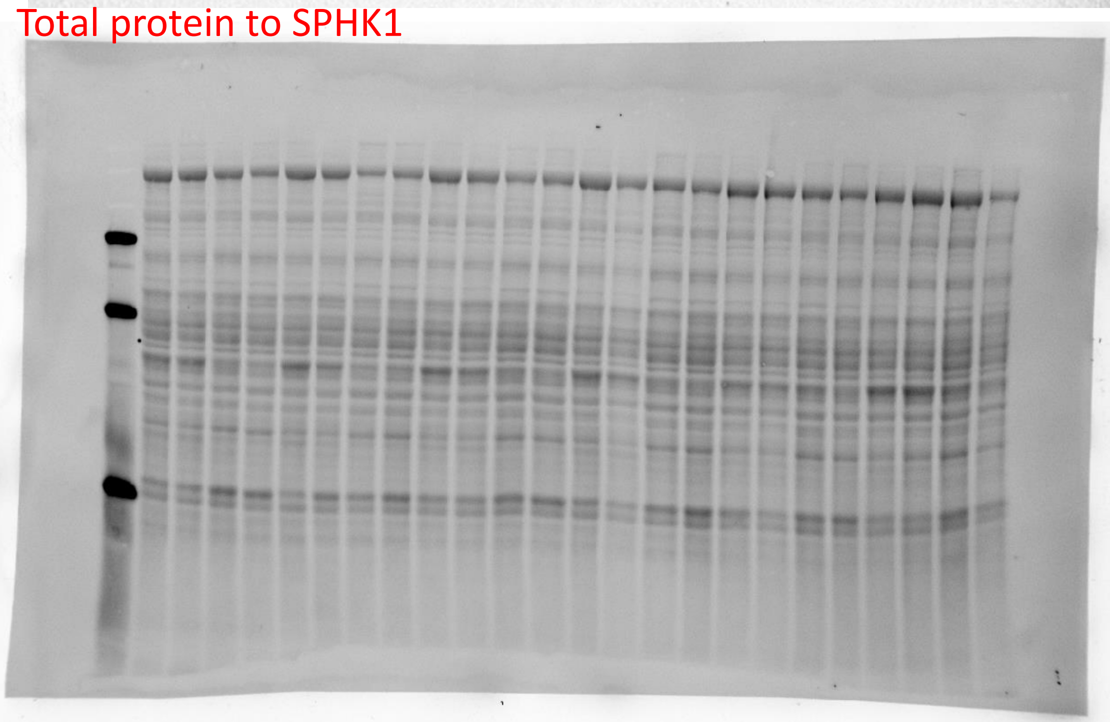
Total protein to SPHK1

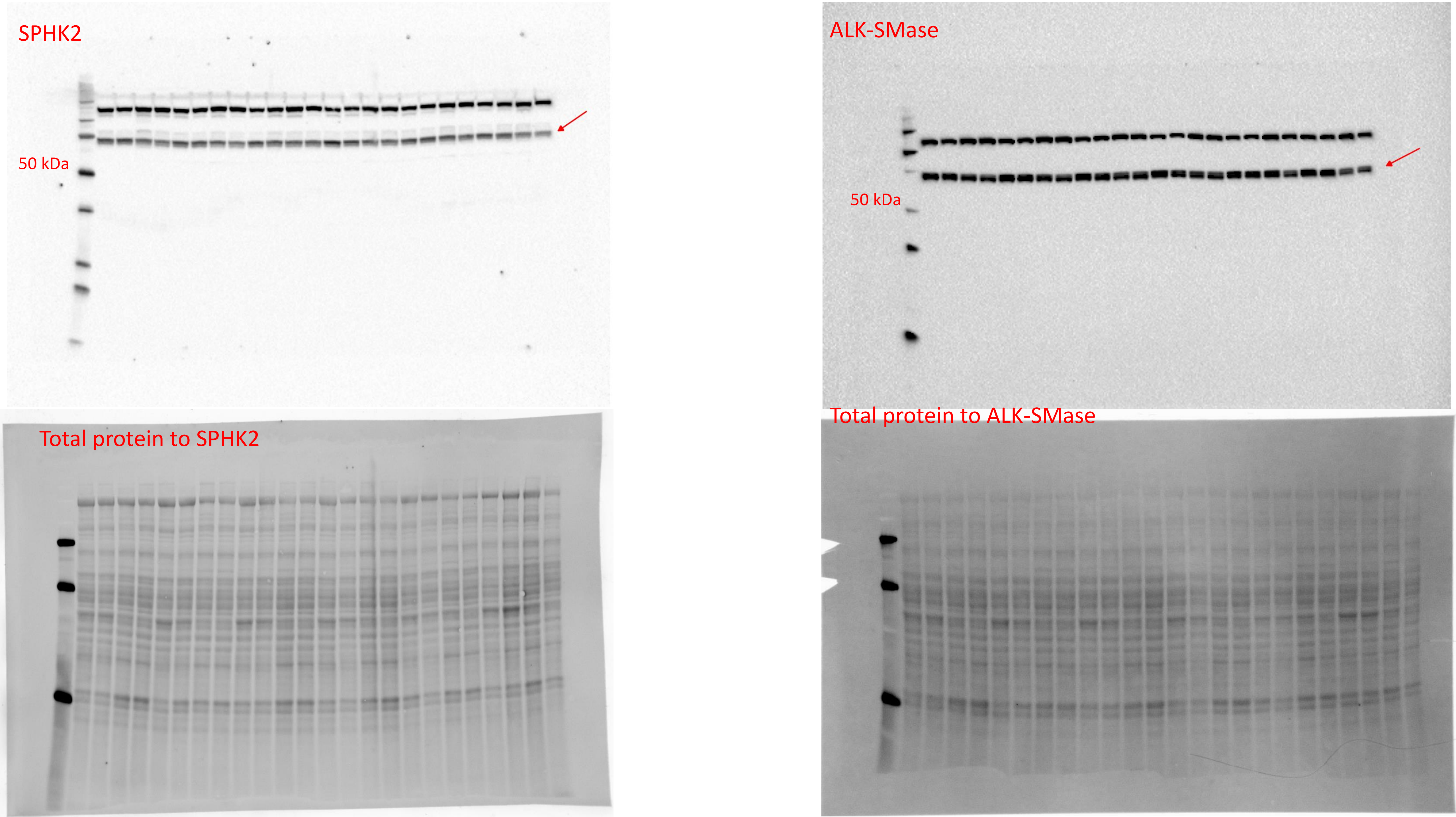
SPHK2
50 kDa
ALK-SMase
50 kDa
Total protein to SPHK2
Total protein to ALK-SMase

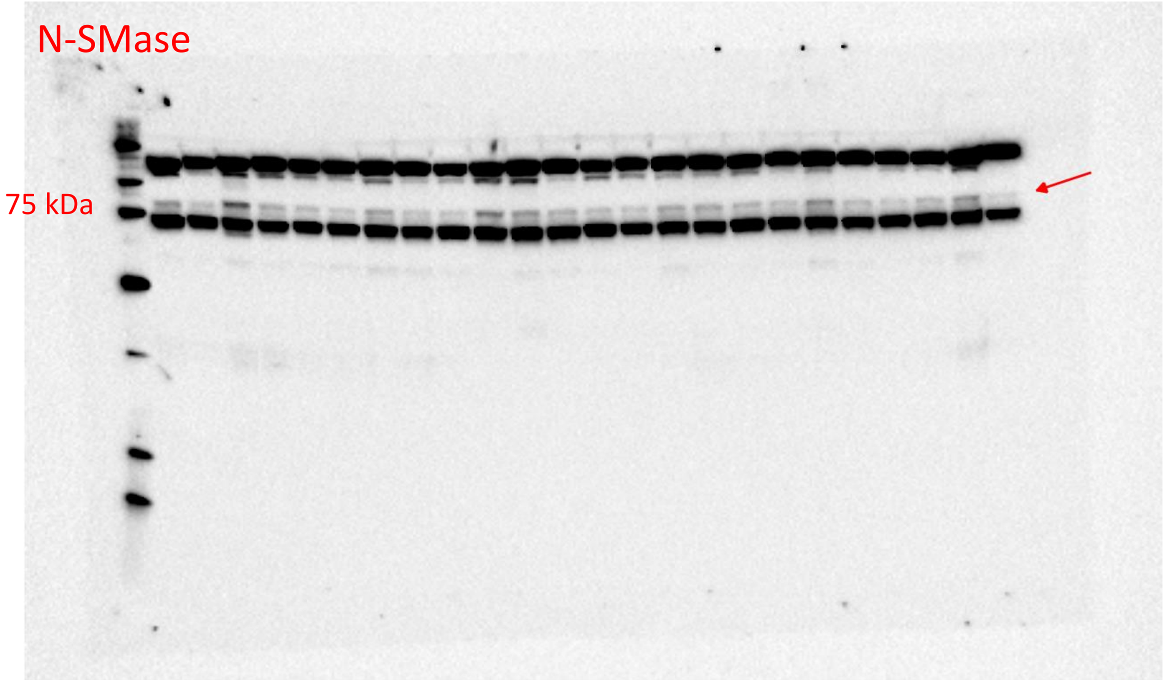
N-SMase
75 kDa

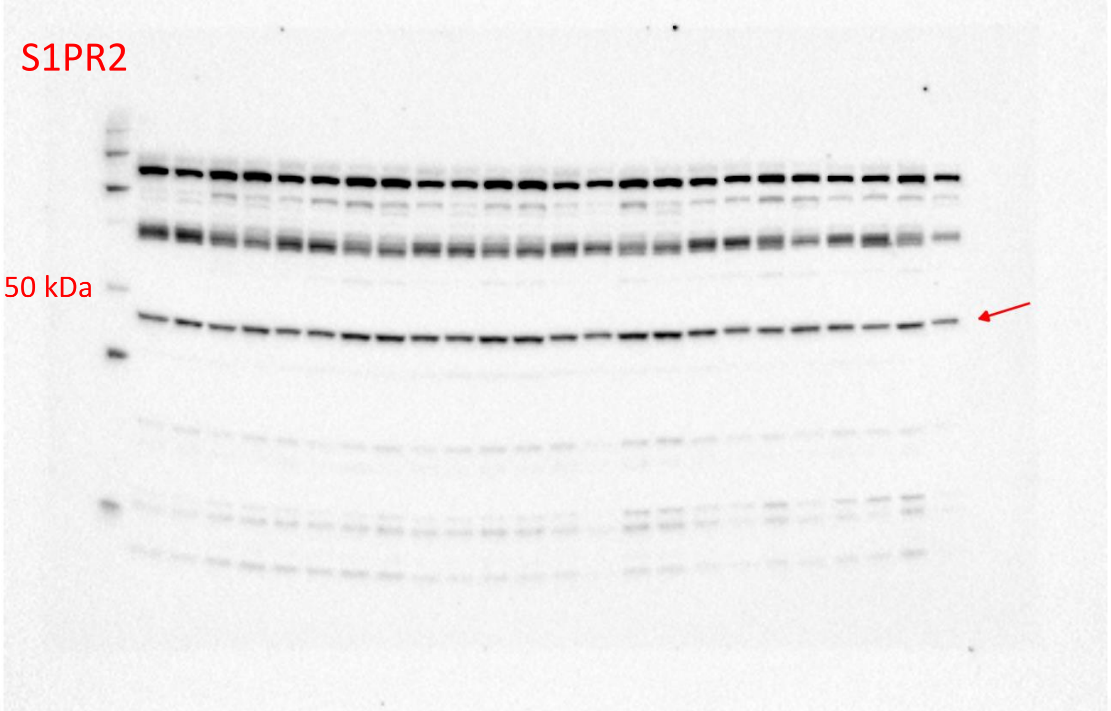
S1PR2
50 kDa

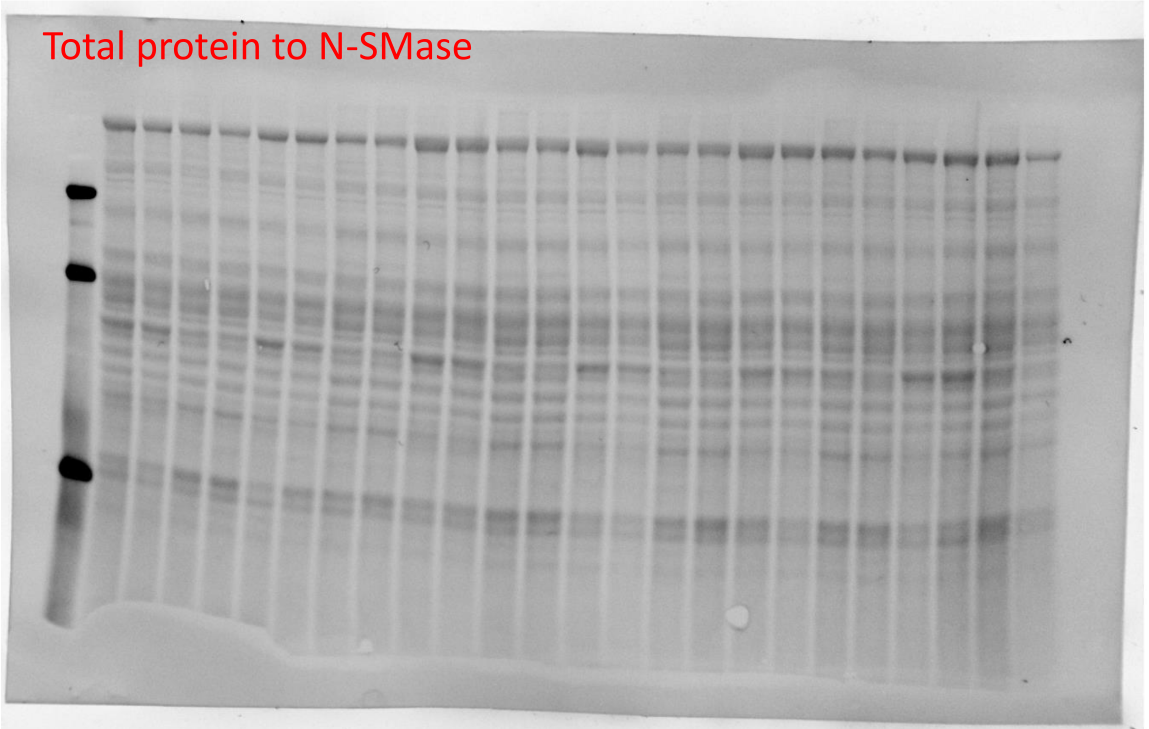
Total protein to N-SMase

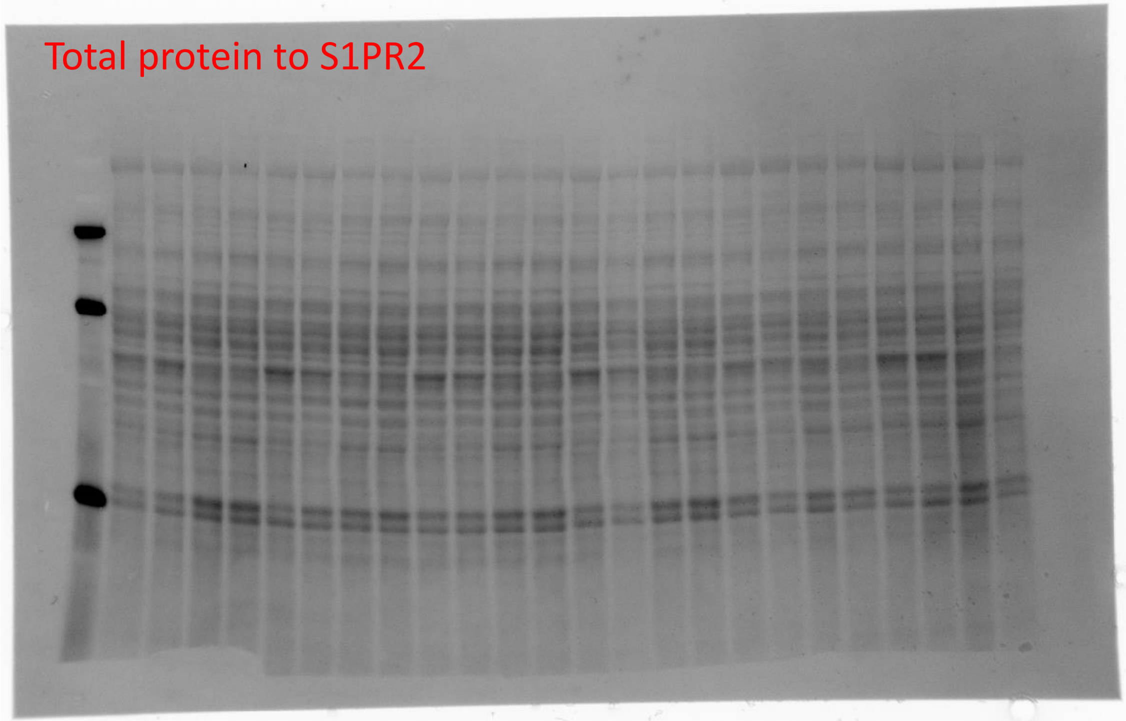
Total protein to S1PR2

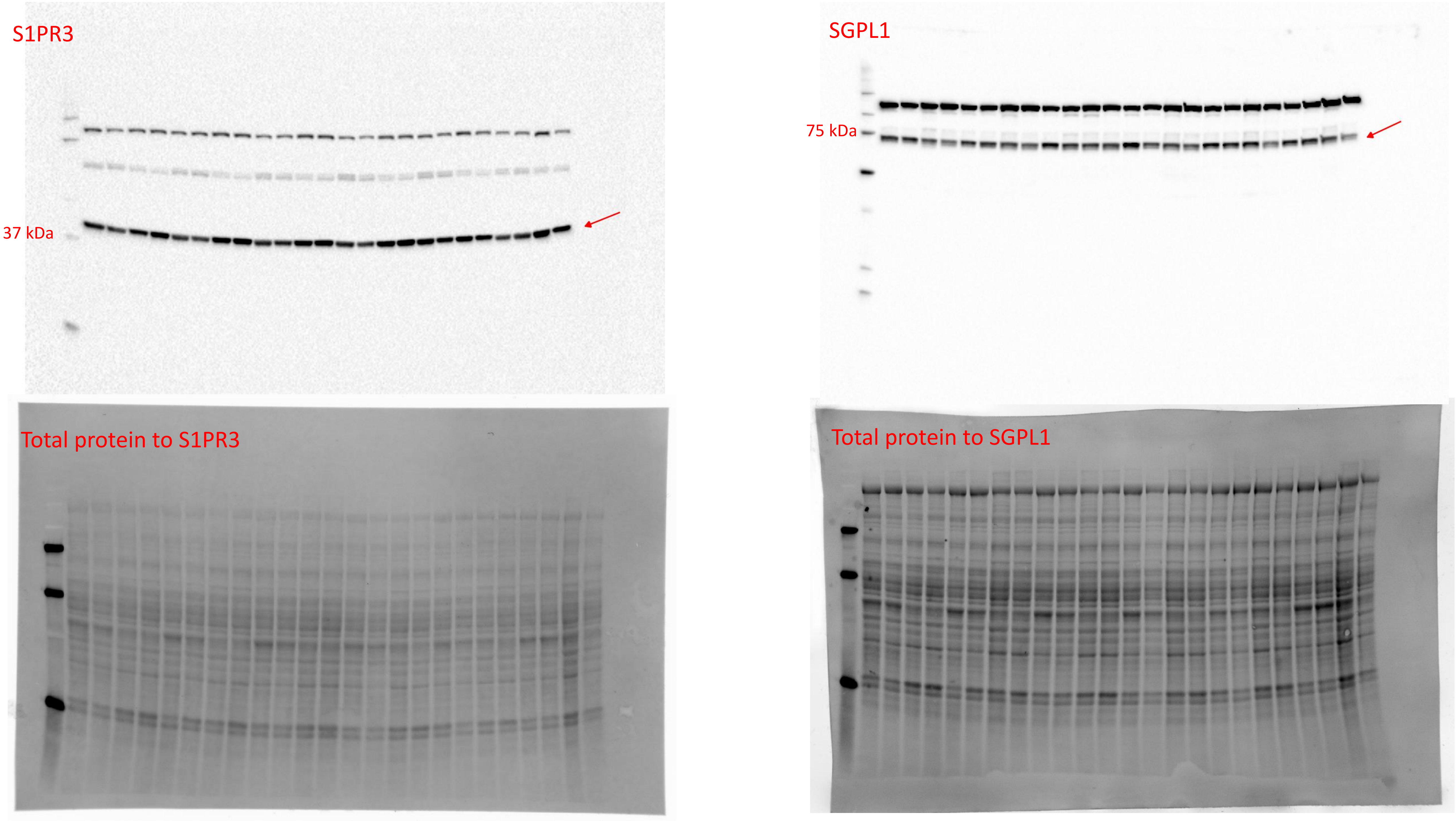
S1PR3
37 kDa
SGPL1
75 kDa
Total protein to S1PR3
Total protein to SGPL1

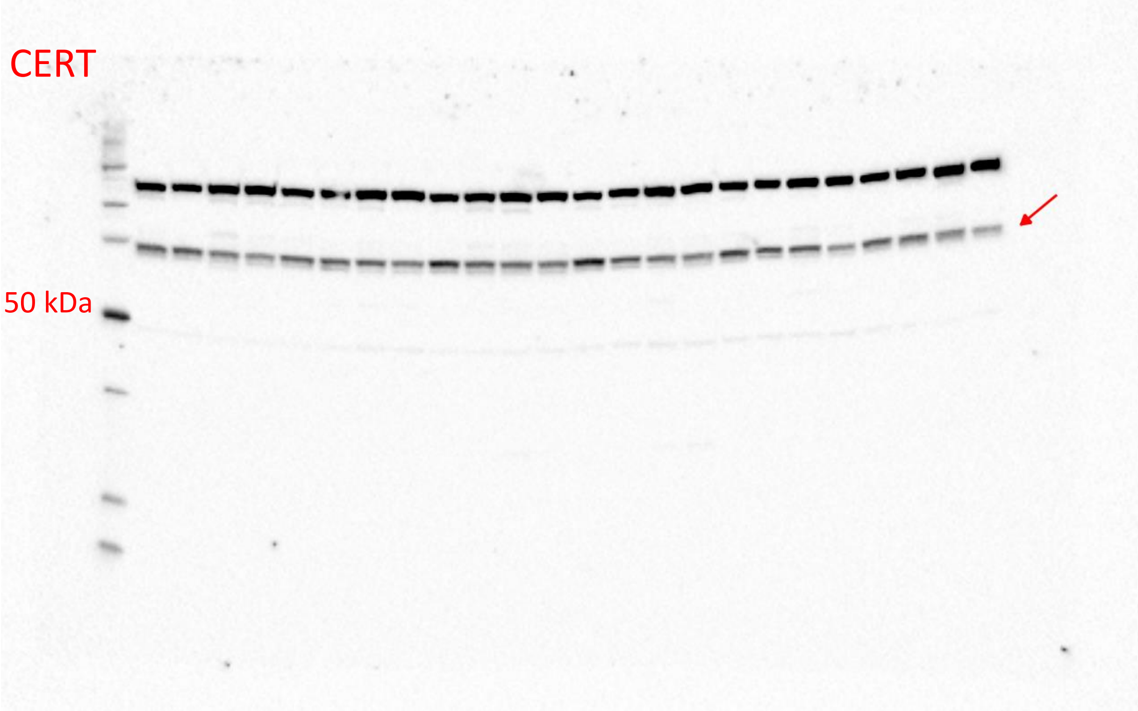
CERT
50 kDa
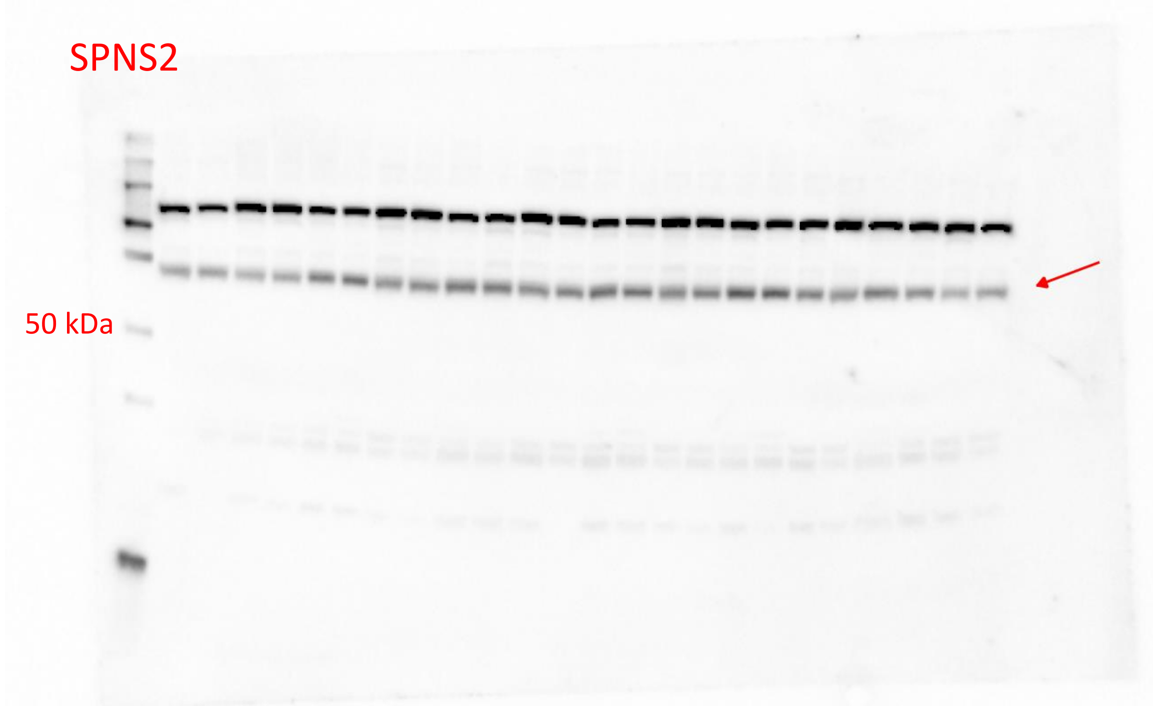
SPNS2
50 kDa
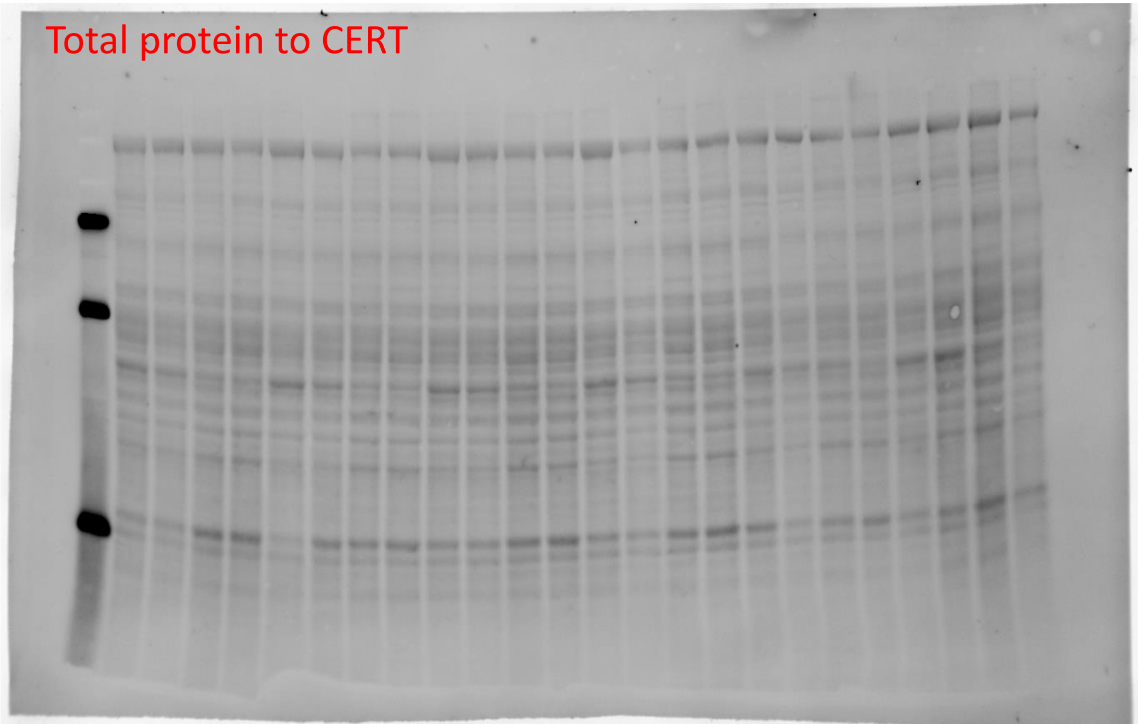
Total protein to CERT
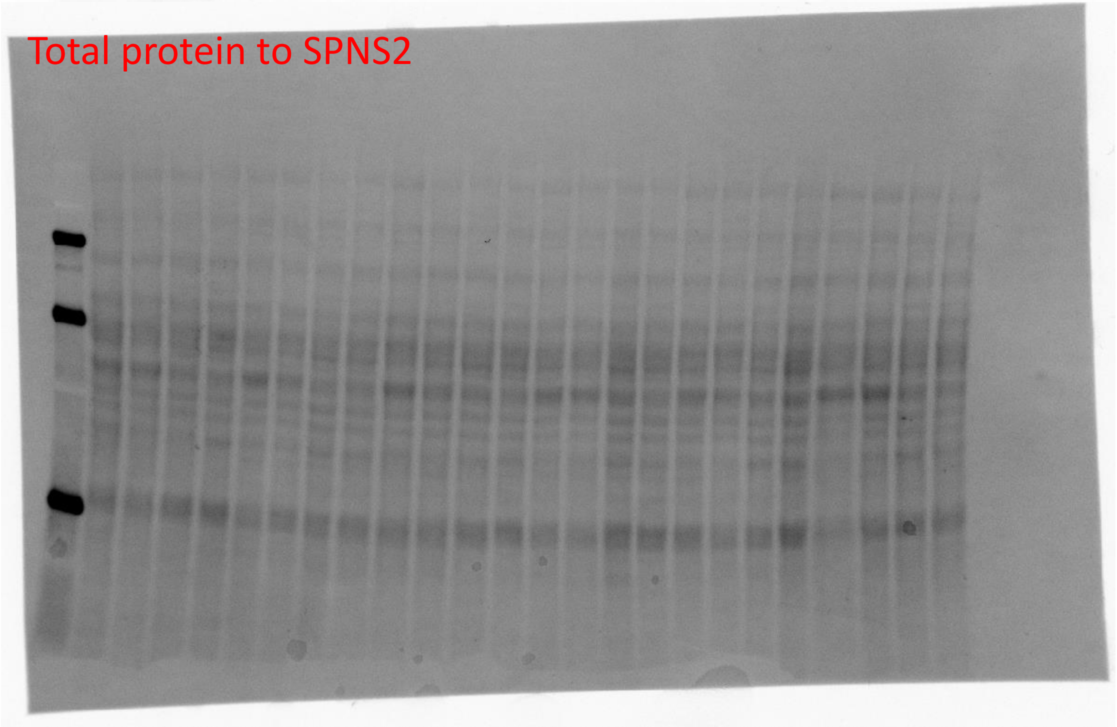
Total protein to SPNS2

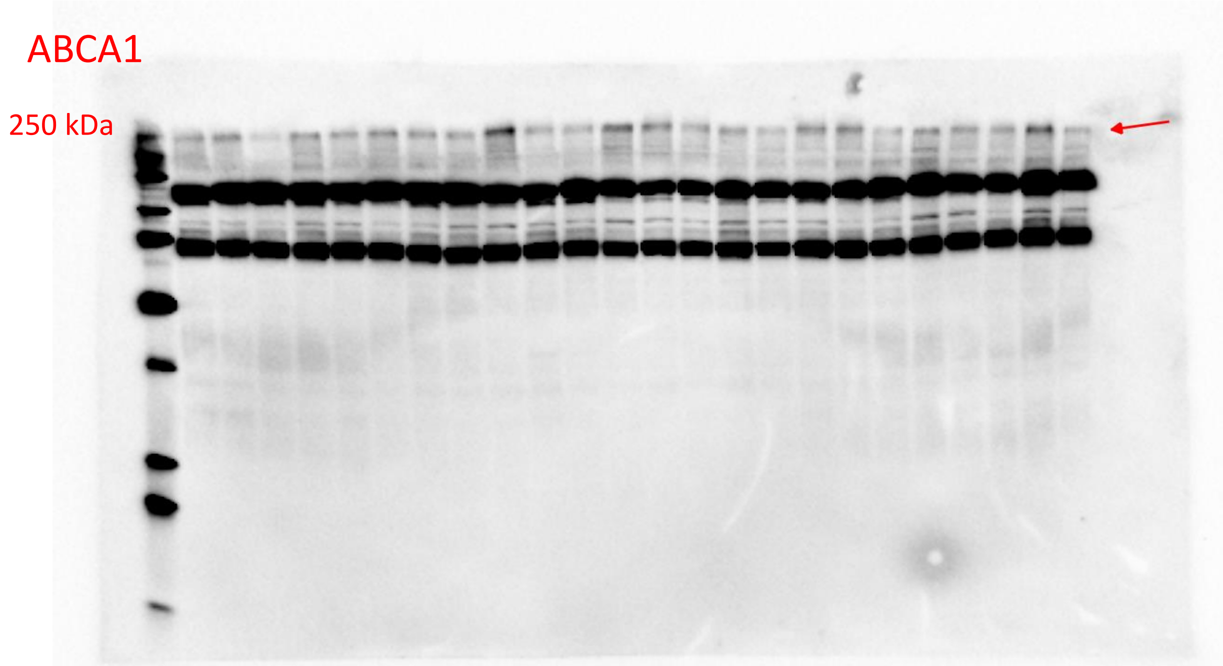
ABCA1
250 kDa

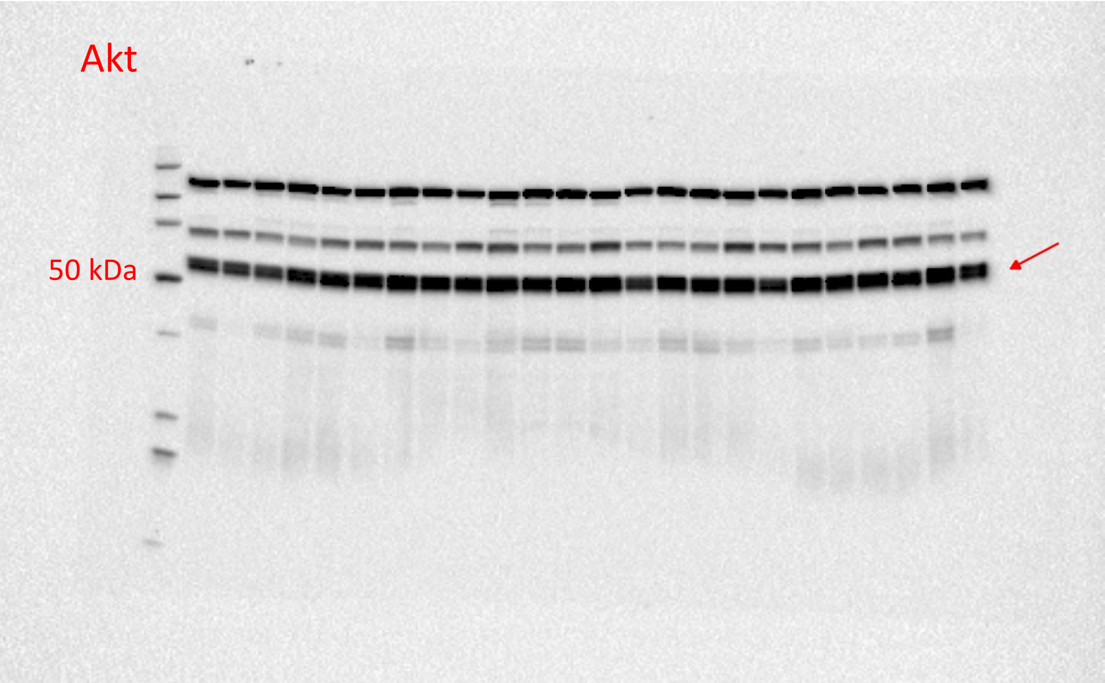
Akt
50 kDa

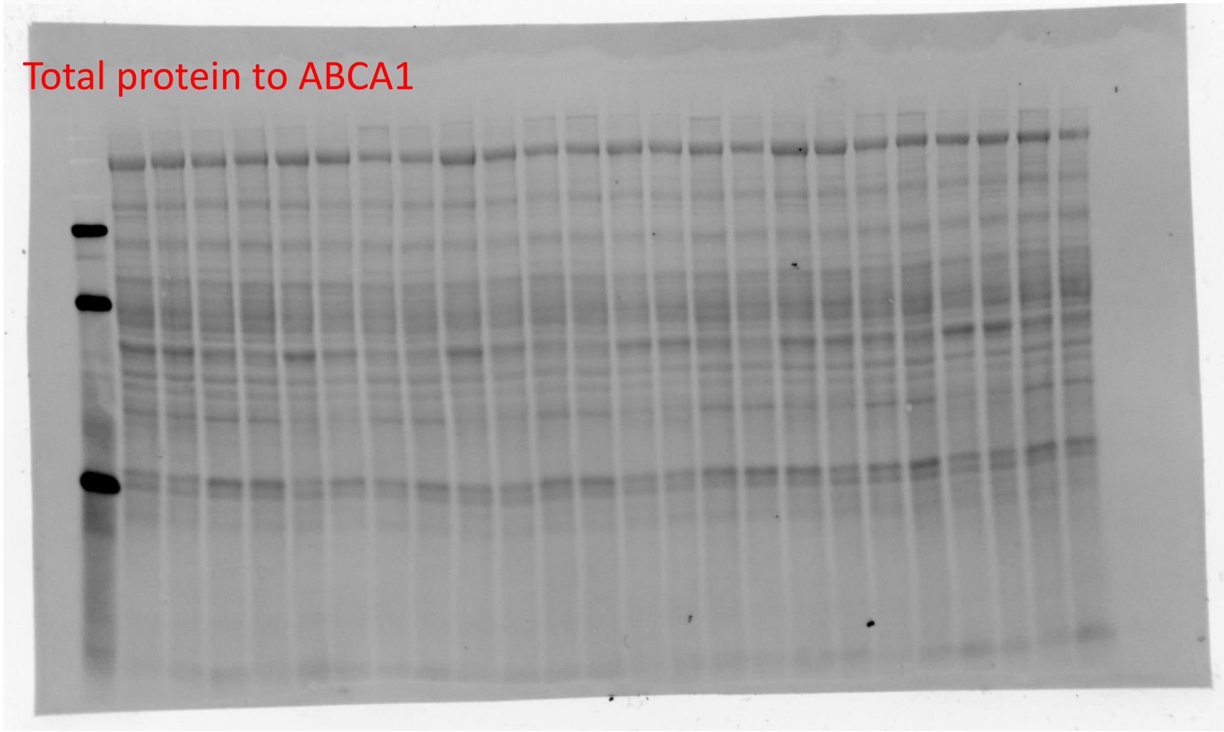
Total protein to ABCA1

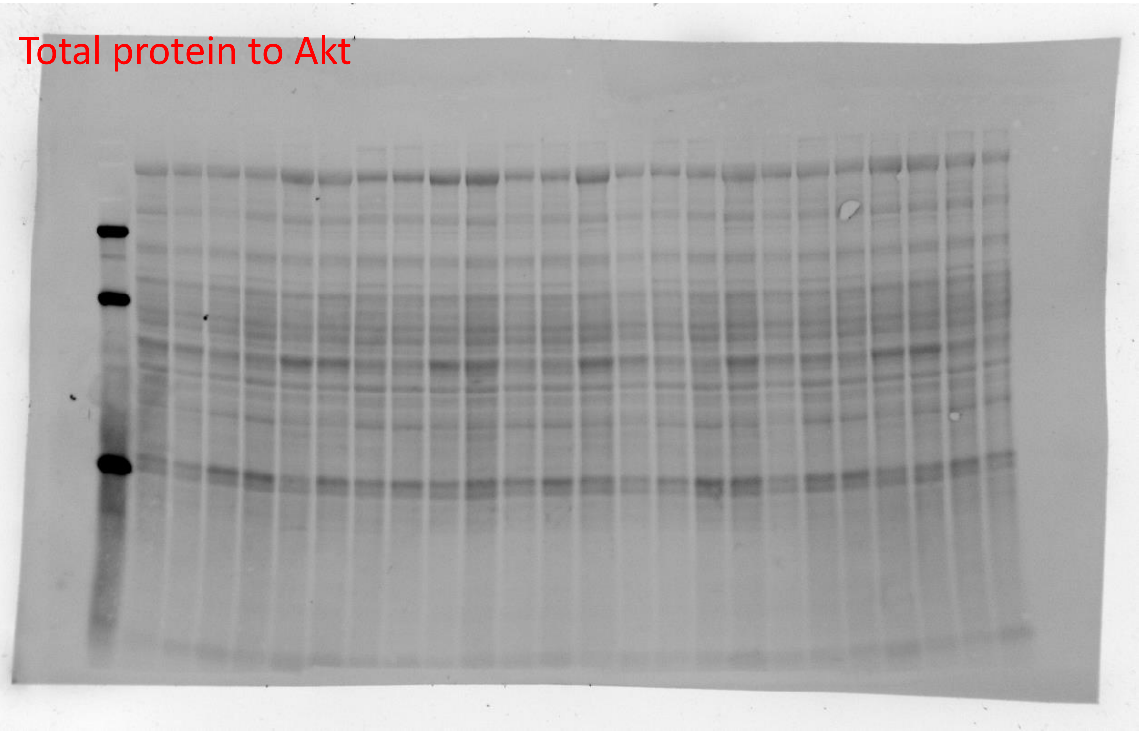
Total protein to Akt

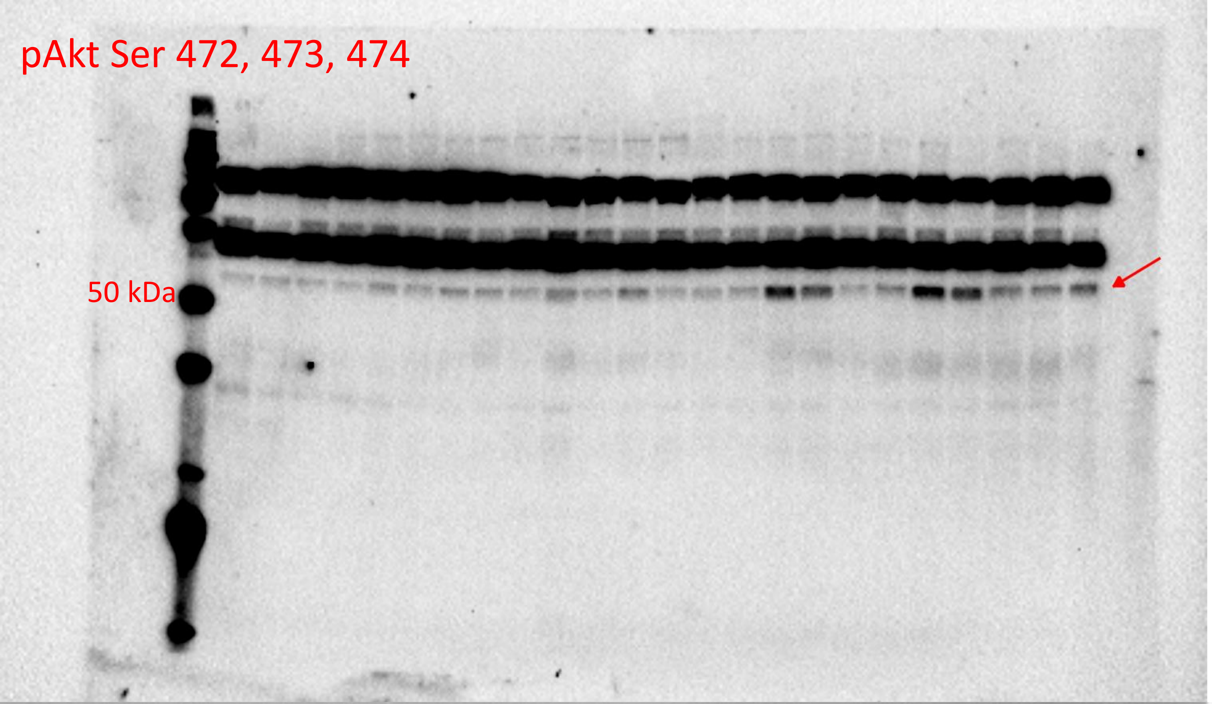
pAkt Ser 472, 473, 474
50 kDa

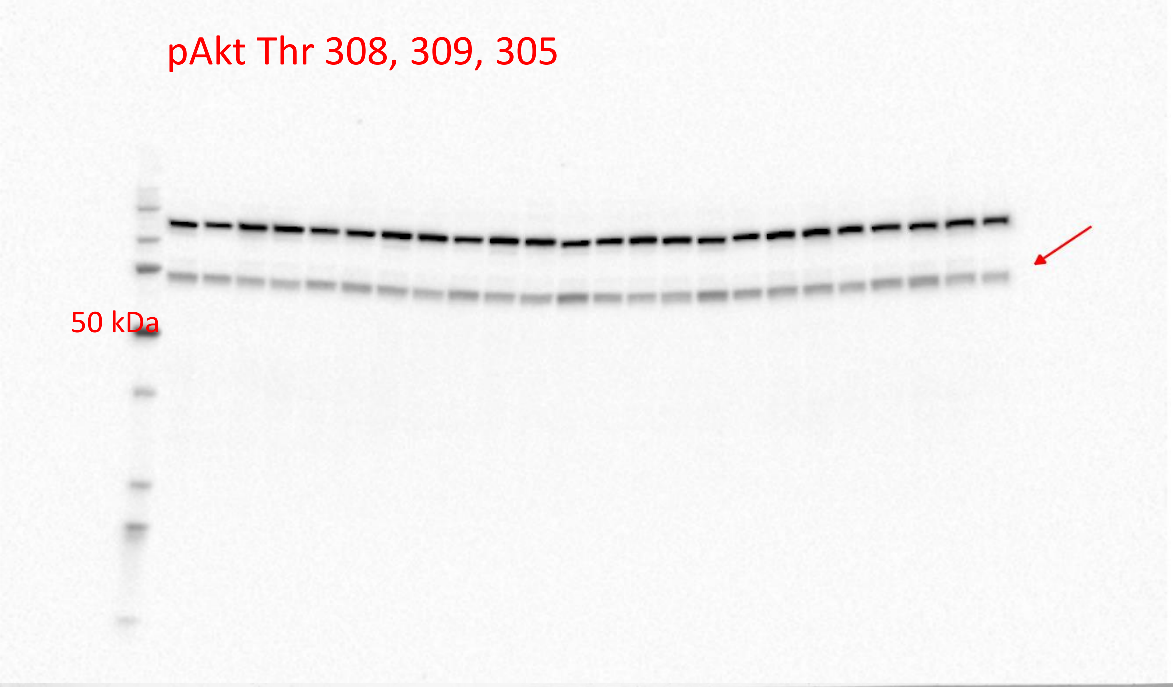
pAkt Thr 308, 309, 305
50 kDa

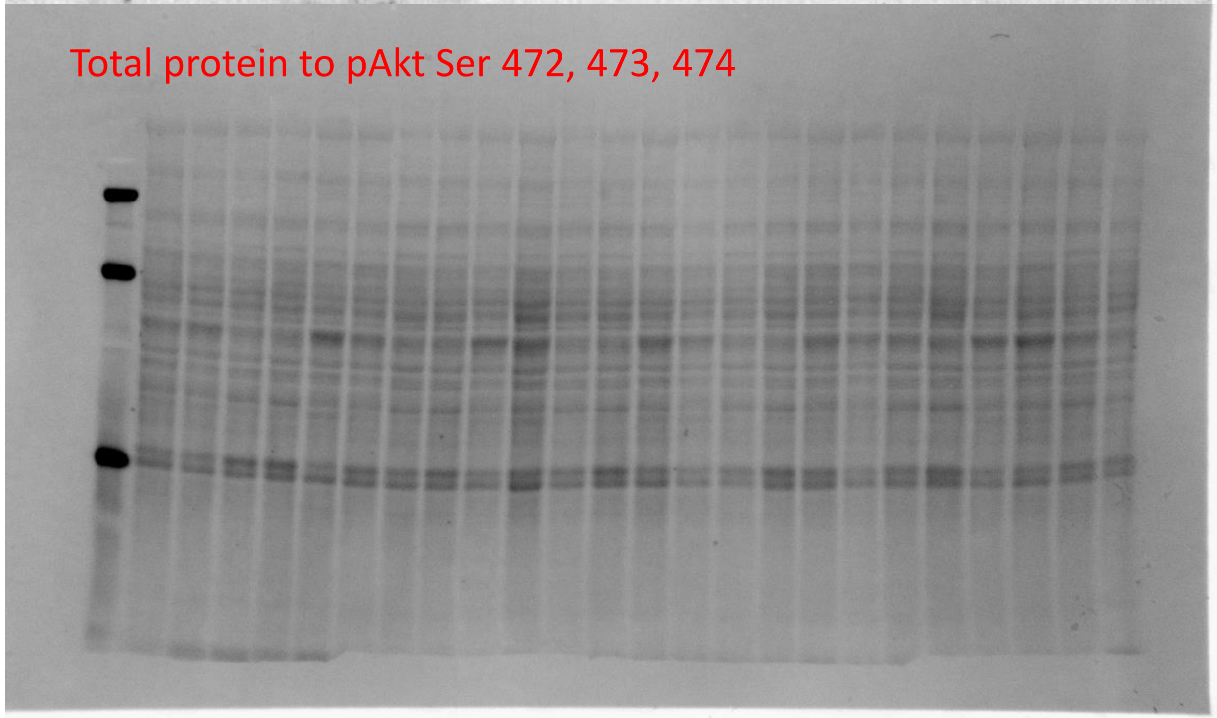
Total protein to pAkt Ser 472, 473, 474

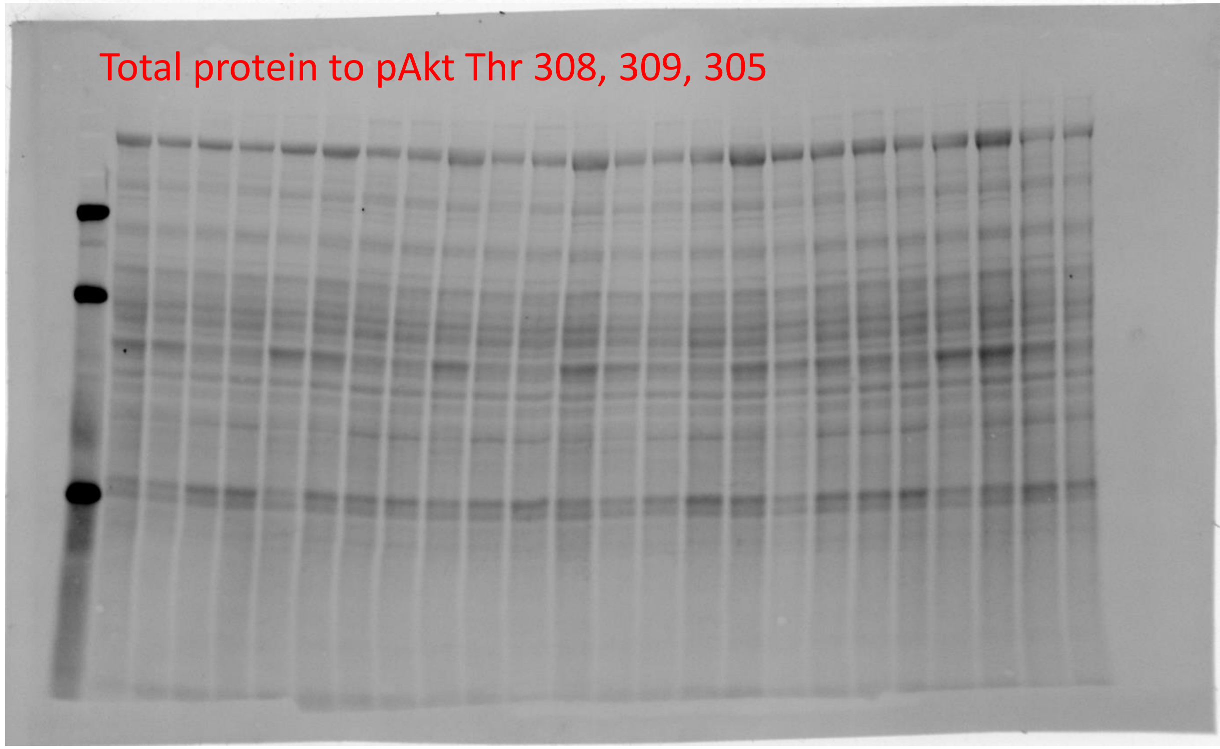
Total protein to pAkt Thr 308, 309, 305

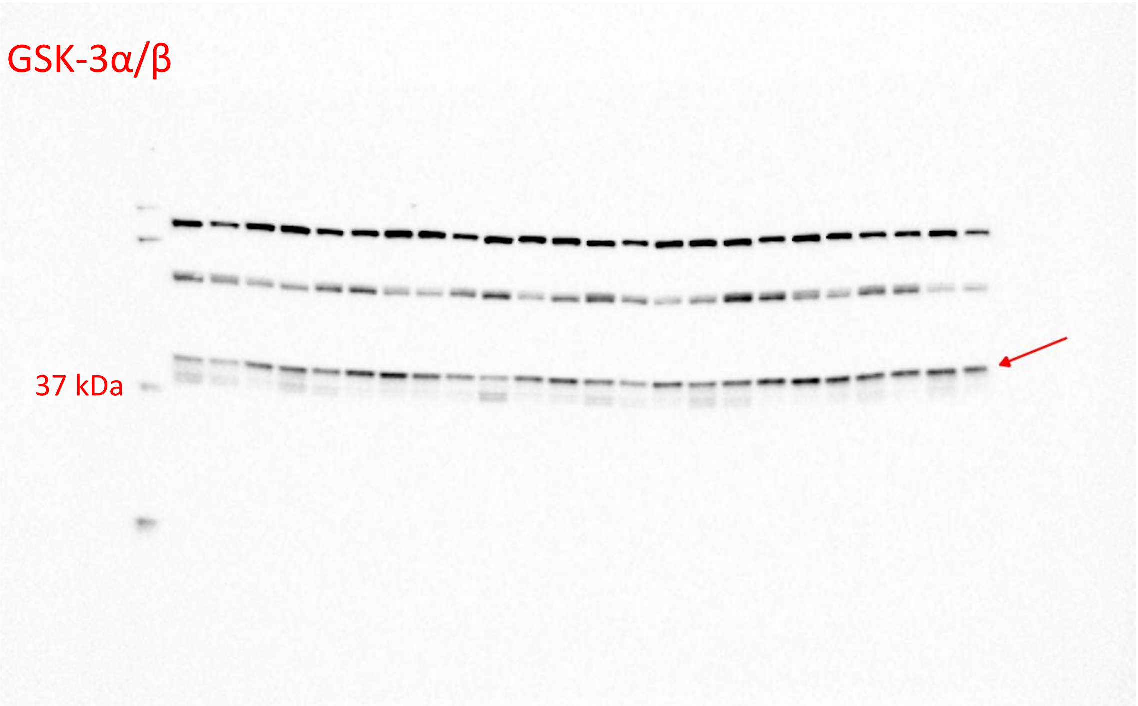
GSK-3α/β
37 kDa

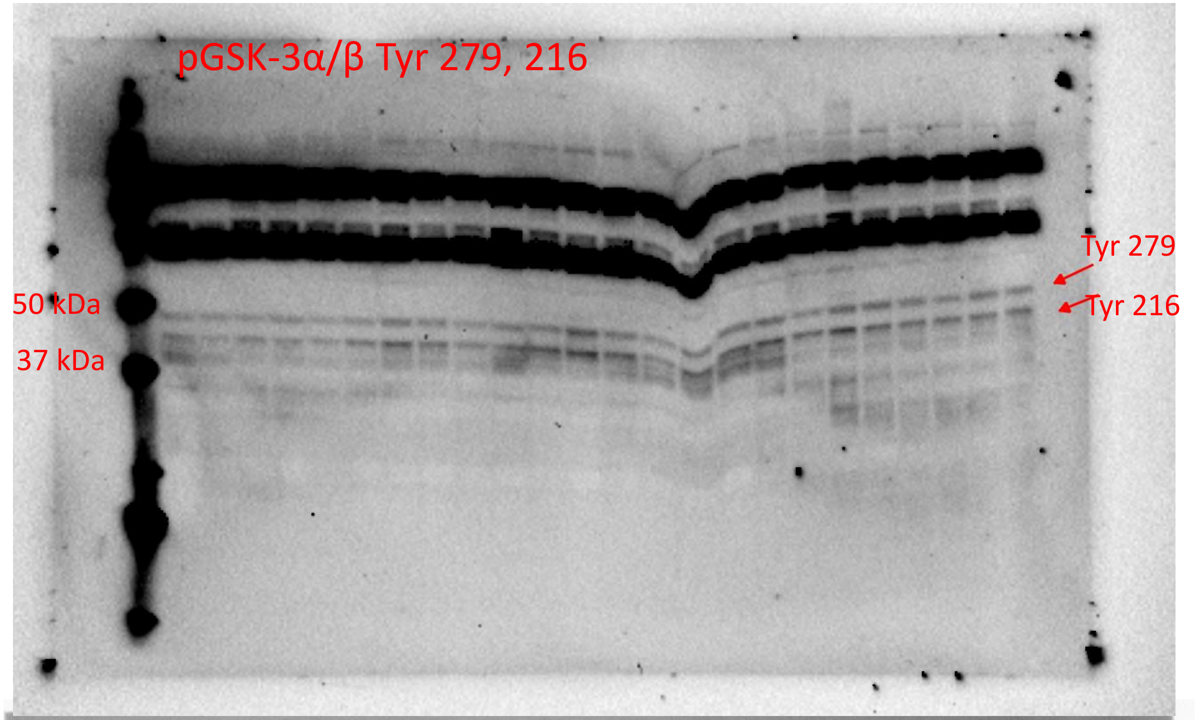
pGSK-3α/β Tyr 279, 216
50 kDa
37 kDa
Tyr 279
Tyr 216

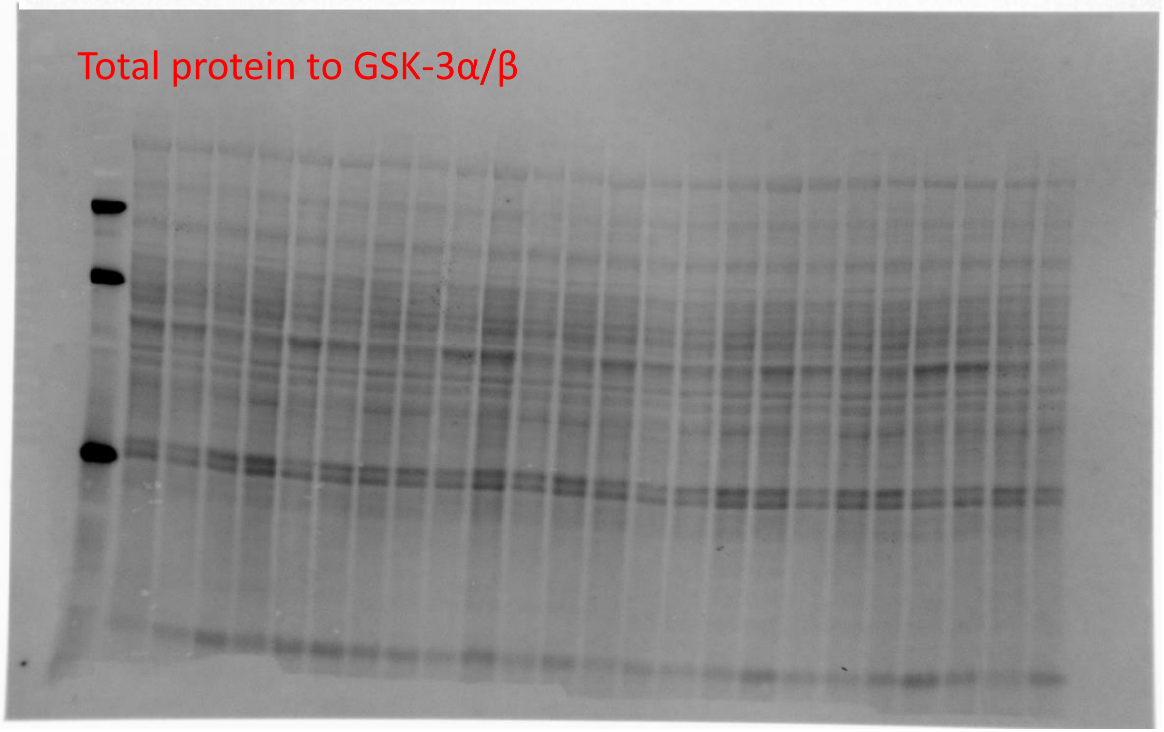
Total protein to GSK-3α/β

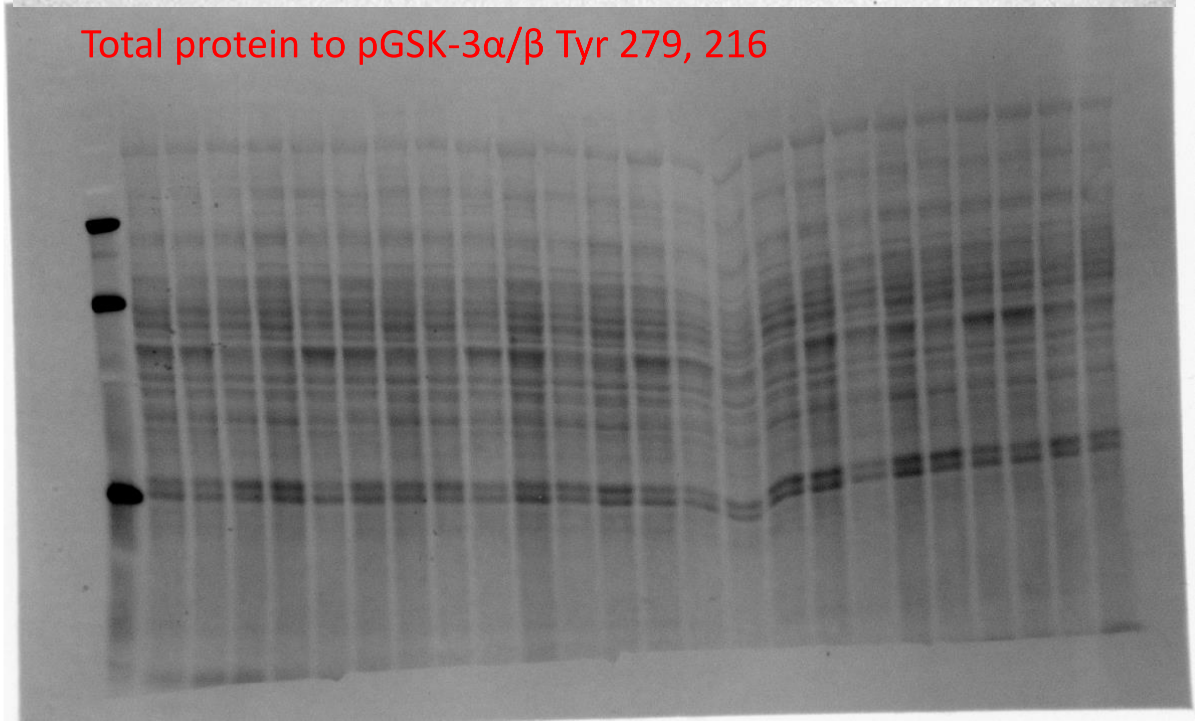
Total protein to pGSK-3α/β Tyr 279, 216

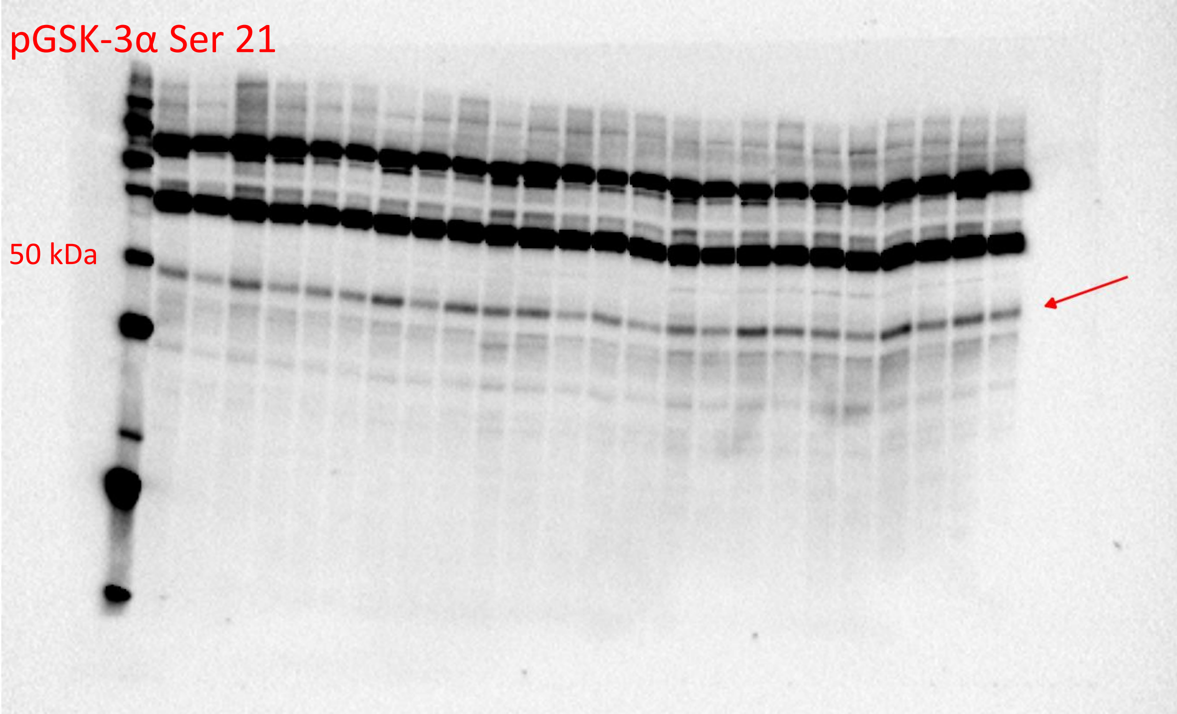

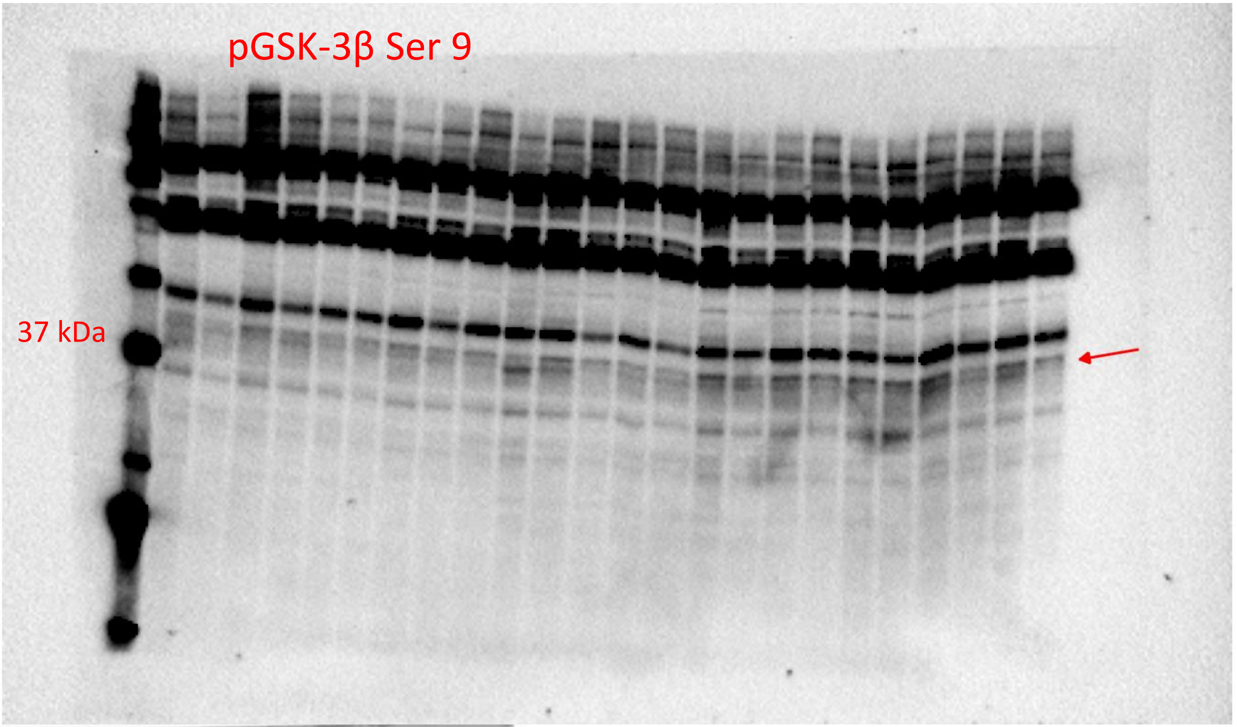

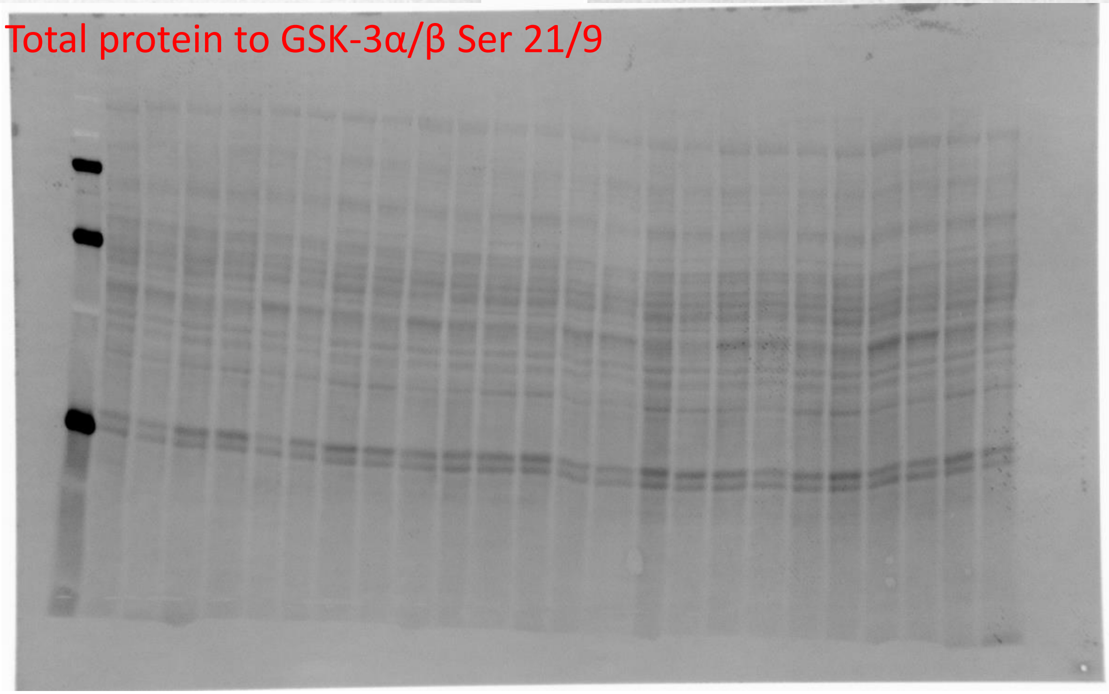

The same blot was used to depict bands for various phosphorylation sites thus total protein image is one for both blots.

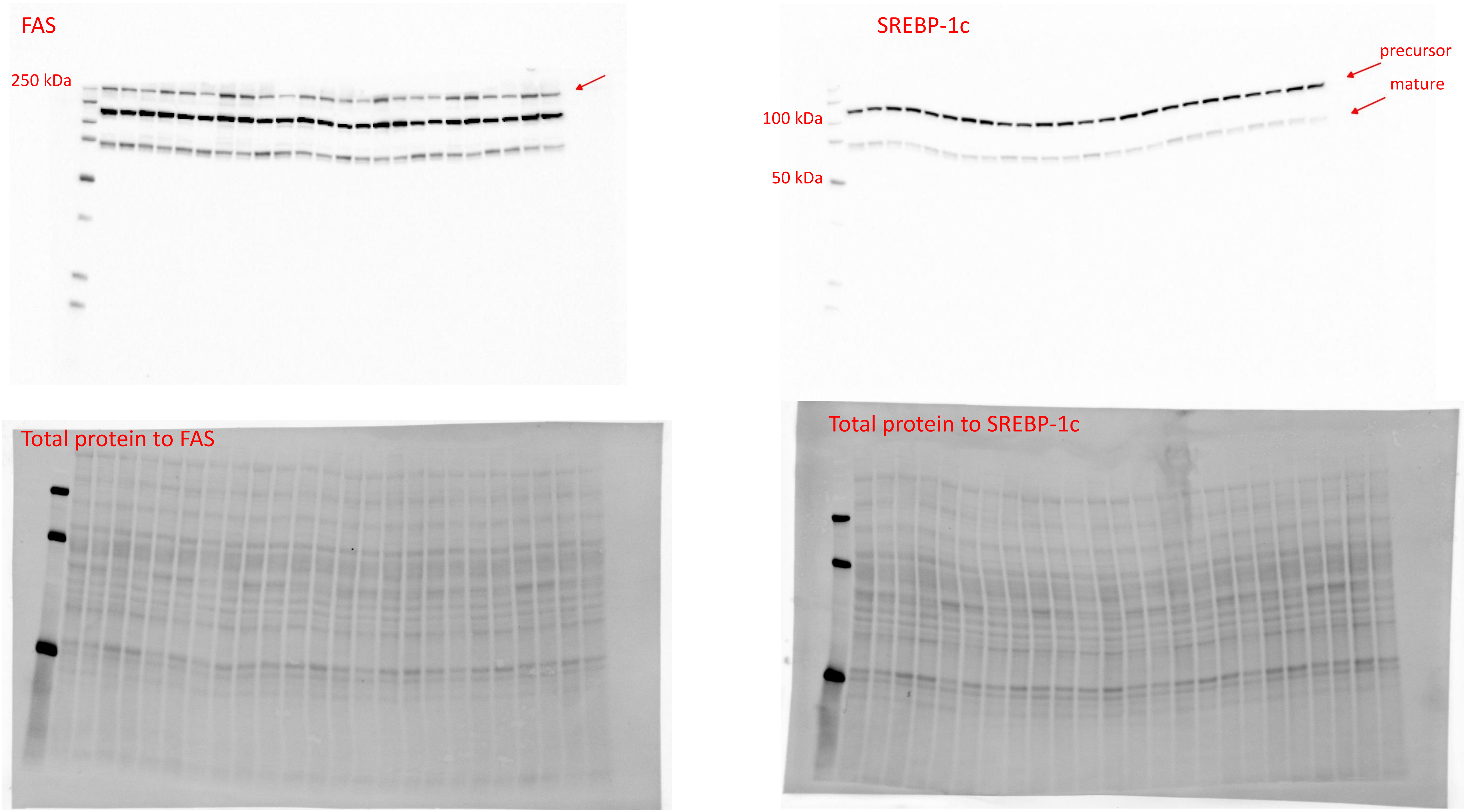
FAS
250 kDa
SREBP-1c
precursor
mature
100 kDa
50 kDa
Total protein to FAS
Total protein to SREBP-1c

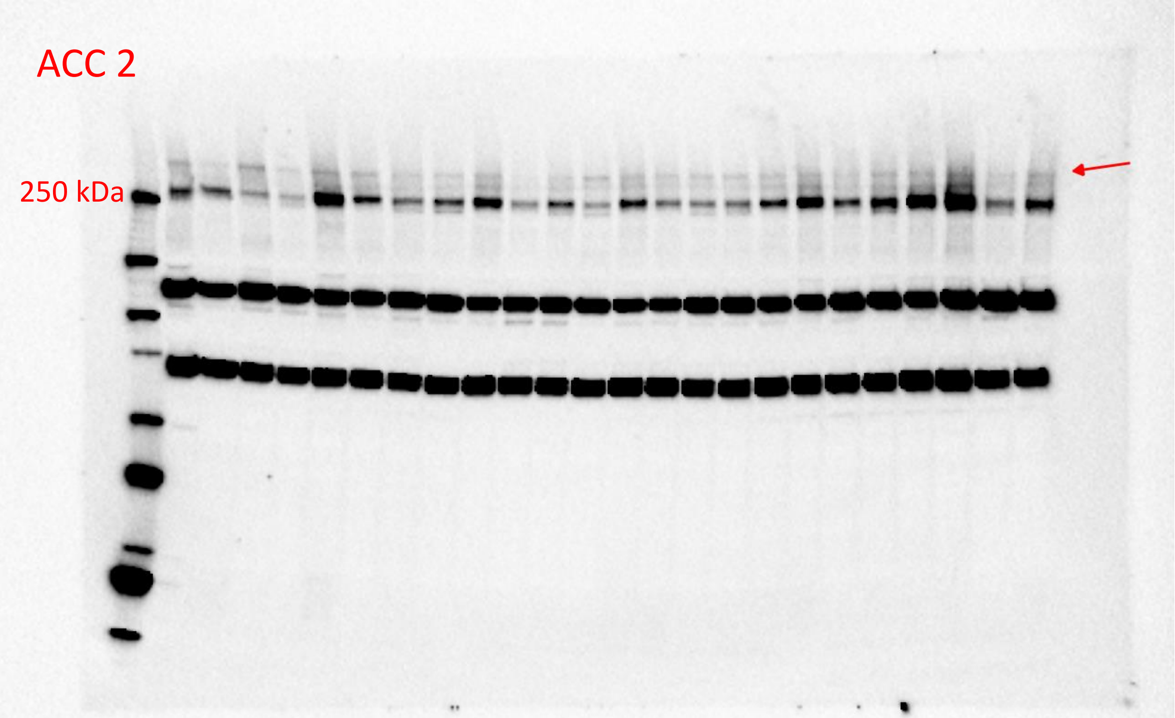
ACC 2
250 kDa

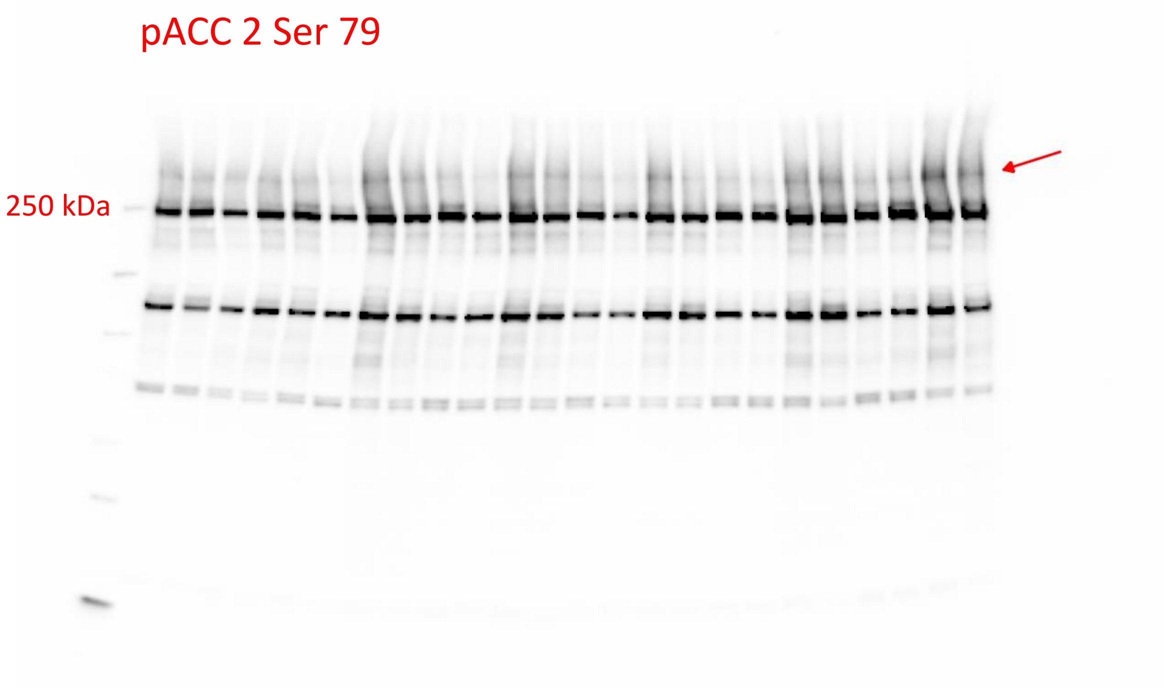
pACC 2 Ser 79
250 kDa

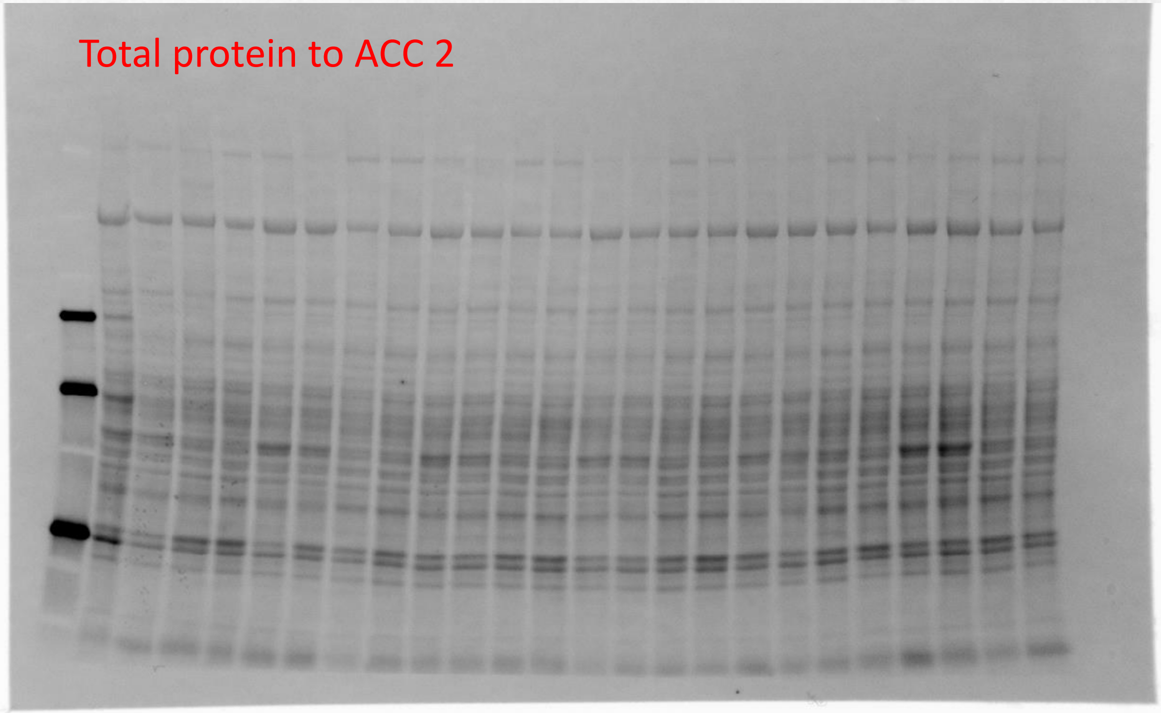
Total protein to ACC 2

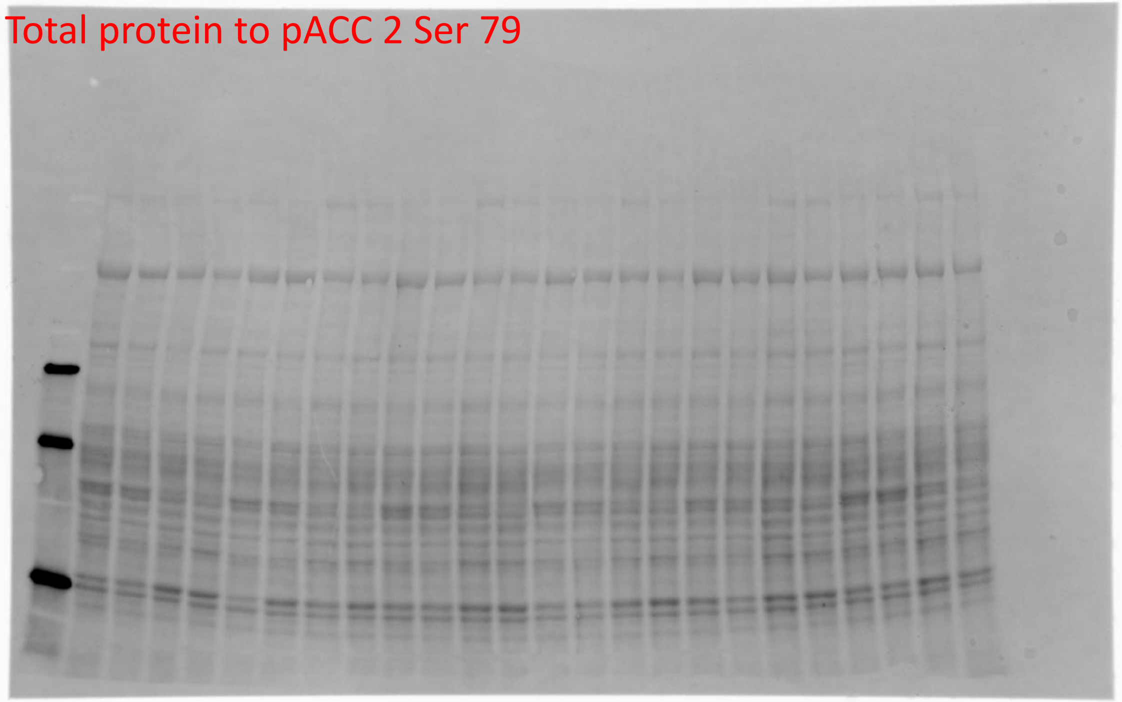
Total protein to pACC 2 Ser 79

PDH

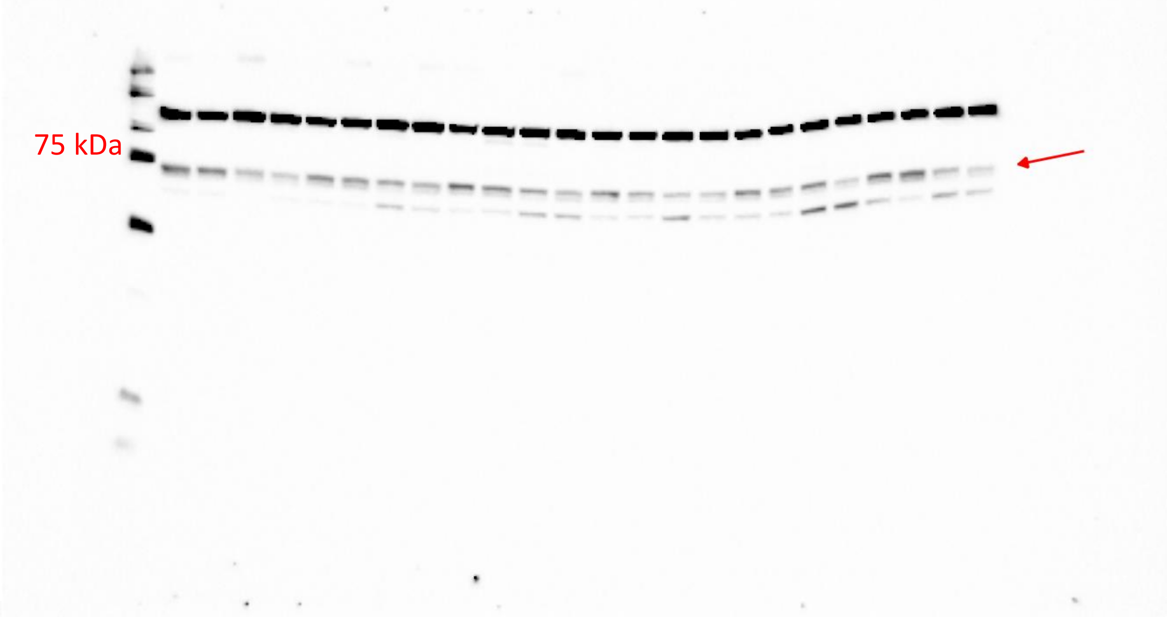

Total protein to PDH

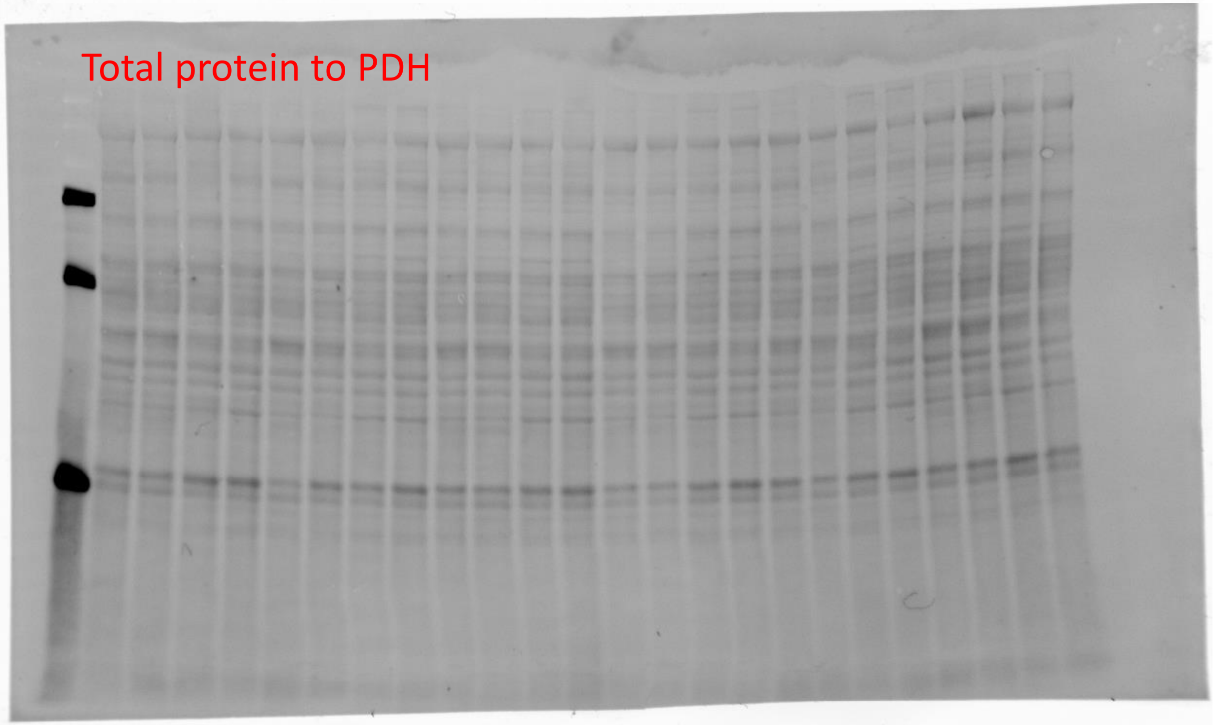

Supplement: Supplementary file 1 [file nutrients-15-04350-s001.zip › nutrients-2628462-supplementary.pdf]
